# Evaluating the Return on Investment of U.S. Army Holistic Health and Fitness Performance Teams: A Matched Difference-in-Differences Study of Readiness and Economic Outcomes

**DOI:** 10.1007/s40279-026-02399-3

**Published:** 2026-02-12

**Authors:** Andrew G. Thompson, Manoj Subedi, Alexander E. Morrow, Chance L. Smith, Kevin A. Bigelman

**Affiliations:** Center for Initial Military Training, United States Army Training and Doctrine Command, 210 Dillon Ave, Fort Eustis, VA 23604 USA

## Abstract

**Purpose:**

Holistic Health and Fitness (H2F) is the United States Army’s largest force modernization initiative aimed at preserving combat power by optimizing soldier readiness across five domains: physical, mental, nutritional, sleep, and spiritual. At the core of this effort are H2F Performance Teams (HPTs): embedded, interdisciplinary subject matter experts, composed of strength and conditioning coaches, athletic trainers, physical and occupational therapists, registered dietitians, and mental readiness professionals. These teams operate within brigades to deliver proactive, preventive, and performance-enhancing interventions that reduce injury risk, accelerate rehabilitation, improve fitness and cognitive performance, and sustain deployability. This evaluation quantified the return on investment (ROI) of embedded HPTs across 56 matched active-duty brigades (28 HPT-resourced, 28 controls), encompassing over 1,000,000 soldiers from fiscal year (FY) 2019 through FY2023.

**Methods:**

A quasi-experimental, presence-based difference-in-differences framework estimated multiyear treatment effects for musculoskeletal injury (MSKI) referrals and profiles, behavioral health (BH) and substance abuse (SA) profiles, Army Combat Fitness Test (ACFT) pass/failure rates, Army Body Composition Program (BCP) noncompliance, and Rifle Marksmanship Qualification (RMQ). Outcome deltas were monetized using validated cost-per-case benchmarks from military/government reports and peer-reviewed studies. A 10,000-draw Monte Carlo simulation, incorporating triangular distributions and a *ρ* = 0.15 Gaussian copula, modeled fiscal uncertainty, interdomain dependency, and force-wide extrapolation.

**Results:**

Despite significantly worse baseline odds pre-resourcing, HPT brigades reversed all major readiness disadvantages by FY2023. MSKI referral odds declined 61% (odds ratio [OR] 1.16 → 0.45), SA profile odds dropped 79% (OR 1.92 → 0.41), and BH > 90-day profile odds fell 44% (OR 1.51 → 0.84). ACFT failure odds decreased 22% (OR 1.05 → 0.82), RMQ expert qualification odds increased 33% (OR 1.21 → 1.60), BCP failure odds decreased 12%, and RMQ failure odds declined 28%. Annually, per brigade, these effects translated to 1363 adverse events avoided and 37,484 duty days restored.

Using domain-specific cost estimates, a 10,000-draw Monte Carlo simulation estimated mean annual cost avoidance of $14.06 M per brigade (95% CI $12.25–16.19 million), with 99.05% of draws exceeding a 4:1 ROI. Duty day restoration and expert RMQ gains added $10.38 million (95% CI $8.15–13.00 million) in readiness value. Combined, annual total economic value reached $24.44 million per brigade (ROI = 8.15:1; 95% CI 7.17–9.27), with force-wide extrapolation yielding $5.28 billion in annual total Army returns. Every $1 invested in HPTs returns $8.15 in value ($4.69 in cost avoidances and $3.46 in readiness improvements).

**Conclusions:**

Embedded HPTs produce robust, statistically significant, multidomain improvements in readiness, performance, and cost efficiency. These estimates exclude long-horizon returns such as retention, disability deferral, or downstream system savings—suggesting total ROI is significantly underestimated. This study indicates HPTs are core readiness infrastructure. Their full-scale implementation is a strategic imperative for modernizing force sustainment and preserving the Army’s most critical asset: the soldier.

**Supplementary Information:**

The online version contains supplementary material available at 10.1007/s40279-026-02399-3.

## Key Points

**Table:** 

*Embedded teams reverse readiness disadvantage and improve multidomain performance.* Brigades resourced with Holistic Health and Fitness (H2F) Performance Teams (HPTs) achieved statistically significant and operationally meaningful improvements across all core readiness domains—despite starting with worse baseline risk odds. By FY2023, HPT brigades outperformed controls in musculoskeletal injury (MSKI), behavioral health (BH), and substance abuse (SA) profiles, while also improving Army Combat Fitness Test (ACFT) pass rates, body composition compliance, and expert-level marksmanship. These results validate the impact of embedded, interdisciplinary teams in restoring deployable strength and improving combat readiness.
*High return on investment and billions in avoided costs.* Monte Carlo simulation estimated a mean annual cost avoidance of $14.06 million per brigade, with 99.05% of draws exceeding a 4:1 ROI. Including readiness gains, total economic value reached $24.44 million annually per brigade (ROI = 8.15:1; 95% CI 7.17–9.27). Force-wide extrapolation across the Active and Reserve Components yields $5.28 billion in annual Army savings—driven by fewer civilian MSKI referrals, reduced attrition, restored duty days, and improved physical and mental readiness. Embedded HPTs offer one of the Army’s most fiscally efficient force sustainment models.
*A scalable blueprint for modernizing human performance*. The HPT model offers a scalable framework for enhancing human performance in high-demand environments. By integrating subject-matter-expert proximity, load management, embedded mental readiness, and recovery optimization, HPTs improve both individual and unit/team outcomes. Beyond the Army, these insights inform injury prevention, performance, and resilience strategies in sport, occupational health, and national security domains.

## Introduction

Global security depends on the sustained readiness of national defense forces [1, 2]. For the United States (U.S.) Army, intensifying operational demands, persistent global engagements, and emerging demographic constraints increasingly challenges the sustainment of soldier performance, health, and deployability. Evolving threat landscapes and the integration of advanced technologies—including surveillance systems, data platforms, and remote sensing—have introduced novel physical, psychological, and cognitive workloads on the Army’s most fundamental resource: its soldiers [3–5]. These pressures span all military occupational specialties (MOSs), placing escalating strain on the foundational systems required to sustain a ready and effective force.

*The readiness crisis*. Musculoskeletal injuries (MSKI), behavioral health (BH) limitations, and preventable attrition collectively erode combat power and force availability. Noncombat injuries remain the single leading health problem in the Military Health System and account for a dominant share of outpatient visits, underscoring their enterprise-level impact on readiness [6–10]. These are not isolated incidents—they are system-level readiness failures responsible for more than 25 million limited duty days annually and more than $3.8 billion in direct medical treatment and lost productivity [7, 9, 11–14]. In the Army, lost duty days are formally tracked through medical “profiles,” which document a soldier’s fitness for duty. A limited duty profile is issued when a soldier’s medical condition restricts their ability to fully perform military tasks [15]. Profiles may be temporary—typically ≤ 90 days for acute injuries—or permanent if a condition is unlikely to resolve. While designed to protect the health of the soldier, accumulated limited duty profiles represent a major readiness burden at the unit and Army enterprise levels [14–17]. Recruitment and retention challenges further constrain the inflow of new talent, with more than 70% of U.S. youth medically or physically ineligible for service due to obesity [18–21], BH diagnoses [22–25], substance abuse (SA) [26–29], or physical deconditioning [30–33]. The compounding effects of these conditions are evident in the extensive time and resources lost to MSKI and attrition [10, 34–36]. The lingering effects of a global pandemic have only intensified these demographic and physiological headwinds, further constricting replacement capacity and elevating the value of prevention and retention [37–39].

The operational consequences of these trends are profound. A brigade combat team operating below authorized strength due to MSKI-, BH-, or SA-related attrition experiences measurable declines in training throughput, deployment capability, and mission success probability [40–42]. Replacing a separated soldier imposes significant costs and time lags—costs that increase with occupational specialty and that can be mitigated by upstream prevention and earlier, effective return-to-duty [13, 34, 43–45]. Untreated or recurrent MSKIs not only impair near-term deployability, but also contribute to chronic pain, osteoarthritis, sleep disturbance, substance use disorders, and elevated risk of depression, anxiety, and post-traumatic stress—burdens that often persist into veteran status to drive long-term disability and healthcare costs [6, 8, 10, 46, 47].

*A structural modernization paradigm shift.* To address these challenges, the U.S. Army initiated the Holistic Health and Fitness (H2F) System—a structural modernization initiative designed to optimize soldier readiness across five interdependent domains: physical, mental, nutritional, sleep, and spiritual [48–50]. H2F integrates doctrine, organizational structure, training, materiel, leadership education, and policy/orders with embedded interdisciplinary teams—Holistic Health and Fitness Performance Teams (HPTs)—in some units. HPTs include strength and conditioning coaches, athletic trainers, physical and occupational therapists, dietitians and nutrition specialists, cognitive performance specialists, program directors, and chaplains. This model shifts the Army from reactive, clinic-based care toward proactive, unit-integrated, individualized, and data-informed interventions across the soldier lifecycle [12, 13, 51–53].

Generic, one-size-fits-all training approaches—historically rooted in standardized physical readiness and wellness paradigms—are increasingly inadequate for addressing the complex and variable demands placed on today’s soldiers. These legacy models often fail to account for variability in age, sex, injury history, physiological capacity, occupational specialty, and recovery potential, all of which influence how service members respond to training and stress [54–56]. Systematic review evidence confirms low fitness, prior injury, female sex, higher body mass index (BMI), and smoking as significant predictors of MSKI in military populations, with risk magnitudes up to 2.15 times baseline [57]. Large-scale military studies demonstrate substantial interindividual differences in response to identical training loads, with some recruits achieving robust gains and others showing minimal or negative adaptations—highlighting the necessity of tailored interventions [56].

Critically, a vast majority of MSKIs are preventable, particularly when upstream interventions such as strength training, load management, early rehabilitation, and recovery optimization are implemented at scale [58–60]. These strategies have demonstrated robust injury prevention efficacy across athletic and military contexts, reducing injury incidence by up to 66% in some populations [58]. Recent evaluations suggest that individualized, strength-based programs improve injury resilience and overall performance more effectively than traditional models. For example, Kyröläinen et al. and Bullock et al. [61, 62] note that optimizing training load, recovery strategies, and periodization significantly reduces MSKI incidence and improves operational outcomes. Moreover, Mikkonen et al. [63] emphasized that sustained strength development plays a critical role in preserving performance under cumulative operational stress, especially in adverse environments. These findings are mirrored in embedded training models that deliver load-managed, autoregulated interventions and demonstrate superior return-to-duty timelines [64]. In practice, U.S. Air Force basic training reduced MSKI rates by ~ 30% in 2 years with embedded performance teams [65]. Embedded athletic trainers have demonstrated substantial value in initial entry training, with odds of returning soldiers to duty up to 10.5 times greater than standard medic care, alongside multi-million-dollar cost avoidance in basic training environments [66].

Importantly, tailoring human performance optimization to the individual not only reduces overtraining and enhances adaptation, it may also mitigate long-term risk factors associated with disability, attrition, and chronic pain [8, 46]. By reducing MSKI burden, improving BH outcomes, and enhancing physical performance, embedded HPTs may offer not only short-horizon readiness gains but also the potential to influence longer-term advantages—including retention, duration of service, disability burden, quality of life, and healthcare expenditures across both U.S. Department of Defense (DoD) and Veterans Health Administration (VA) systems [8, 13, 45, 67–70]. The financial implications are substantial: MSKI-related disability compensation and veteran healthcare costs now exceed billions annually [9, 45, 68, 71, 72]. By proactively addressing these risks, HPTs may deliver exponentially compounding value over the lifecycle of the soldier—preserving deployability, improving operational effectiveness, extending service years, and reducing downstream cost burdens borne by the Army and the U.S. taxpayer alike. Together, this evidence supports investigating the strategic shift toward embedded, interdisciplinary HPTs as an operational necessity for modern force sustainment.

*Need for rigorous evidence:* Unlike previous wellness campaigns or isolated programs, H2F is structural—it redefines how the Army generates and preserves combat readiness. Yet, like all modernization initiatives, implementation alone is insufficient. H2F must demonstrate return on investment (ROI) in terms that are both operationally meaningful and analytically rigorous. This includes reductions in injury and attrition, improvements in combat readiness, and measurable cost efficiency. As arguably the world’s largest human performance optimization initiative, impacting nearly 1 million soldiers annually, H2F provides an unprecedented opportunity to model structural ROI at scale. Embedded HPTs may not be a marginal program investment—instead, they may represent a potential force-wide transformation in how human capital is developed, protected, and sustained. Their impacts could span prevention, recovery, performance, and retention—unifying multiple readiness domains into a cohesive operational advantage. Quantifying the real-world ROI of this model is therefore essential, not only to inform continued implementation, but also to ensure evidence-based modernization of the Army’s force sustainment enterprise.

Accordingly, this study rigorously evaluates the ROI of embedded HPTs within the U.S. Army using a presence-based, intent-to-treat, quasi-experimental, difference-in-differences (DiD) design. Specifically, it examines whether brigades resourced with embedded HPTs demonstrate superior readiness, performance, and economic outcomes compared with matched control brigades across a 5-year period (FY2019–FY2023).

This evaluation addresses two core analytical questions:

*Q1a–e.* Do HPT-resourced brigades exhibit significantly different outcome trajectories, odds ratios, or performance shifts compared with matched controls? What is the magnitude and direction of these differences, and how might they scale across the force?

*Q2a–b.* Do these differences manifest in distinct temporal patterns over time, as reflected in group-by-year interaction effects?

*Hypotheses*. We hypothesized that brigades resourced with embedded HPTs would demonstrate significant and sustained improvements in readiness, performance, and fiscal outcomes compared with matched control brigades, independent of baseline differences. These effects were expected to emerge over the FY2020–FY2023 evaluation window and would be observable across both mean trends and probability distribution. Specifically:

- H_1_ Readiness: HPT brigades would exhibit statistically significant reductions in adverse outcomes, including MSKI referrals, MSKI profiles > 90 days, BH profiles, BH profiles > 90 days, and SA profiles, compared with controls. These reductions would be supported by favorable group-by-year interaction effects in factorial analysis of variance (ANOVA) models and shifts in baseline odds ratios by FY2023.
- H_2_ Performance: HPT brigades would demonstrate statistically significant improvements in physical performance outcomes, including increased ACFT pass rates and expert RMQ, as well as greater reductions in BCP and RMQ failure odds, relative to matched controls. Differences would be confirmed via both longitudinal group-by-year interactions and within-year Chi-squared contrasts in terminal year comparisons.
- H_3_ Economic: Observed outcome differences would translate into measurable cost avoidance and monetized readiness gains, with HPT brigades producing a higher total economic value (TEV) per brigade. We hypothesized that all Monte Carlo simulation estimates would exceed breakeven ROI thresholds, with > 95% of draws surpassing the 2:1 ROI benchmark.

This hypothesis structure reflects a scalable, conservative, intent-to-treat analytic framework that rigorously evaluates the readiness, performance, and economic impact of embedded HPTs in operational Army brigades. The study also contributes to a growing evidence base across sport, corporate, and allied military settings where embedded human performance models have demonstrated similar multidomain benefit patterns [73, 74].

## Methods

Randomized controlled trials are the gold standard for establishing causality between interventions and outcomes. However, when randomization is infeasible, unethical, or operationally constrained—as is often the case in large-scale military system changes—rigorous quasi-experimental designs offer valid and actionable alternatives [75–78]. When system-wide initiatives are implemented through policy-driven prioritization (rather than random assignment), quasi-experimental methods must be used to estimate causal effects with appropriate rigor [77, 79–81]. In this observational evaluation, HPTs were not randomly assigned. Instead, Army leadership prioritized resourcing on the basis of operational demand, known readiness challenges, and force modernization criteria [50, 82]. These nonrandom treatment assignments necessitated careful design of analytic controls, including matched comparator brigades and longitudinal DiD models to account for baseline imbalances.

Matched control brigades were selected on the basis of structural similarity: comparable mission roles, unit size, MOSs, and regional climate. Most brigades were drawn from combat and combat-support formations (e.g., infantry, engineers, field artillery), with HPT and control brigades operating from separate installations to prevent cross-contamination and to mitigate shared access to local medical resources. While both groups could engage in Army-wide H2F initiatives (e.g., educational programs, digital content, fitness training), only the HPT-resourced brigades received embedded interdisciplinary teams integrated into daily operations. This design allows for the estimation of treatment effects under real-world implementation constraints, using matched controls and temporal trend comparisons to strengthen internal validity [83].

### Data Access

All outcome data were extracted from official U.S. Army and DoD systems of record (see Supplementary Table 1, Online Resources for full list of HPT ROI metrics, data sources, timelines, and details). Because the analysis used deidentified, retrospective operational data, no written informed consent was required. Per 45 CFR §46.102(l), evaluations of authorized operational programs conducted to support national security are not considered human subjects research and are exempt from Institutional Review Board (IRB) oversight [84]. The evaluation was conducted under the authority of Department of the Army Execution Order 149-19, which established H2F as an official modernization effort and directed the tracking and reporting of ROI metrics to inform strategic readiness decisions [50].

All reported outcomes had complete brigade-level data from FY2019 through FY2023. Access to the underlying dataset is restricted due to operational security. However, external data access requests may be considered through formal channels. Requests for data access may be submitted to the U.S. Army Center for Initial Military Training (CIMT) Public Affairs Office (usarmy.jble.tradoc.mbx.cimt-pao@army.mil) and must include justification of need, institutional review, and secure information handling protocols.

### Participants

All soldier data were deidentified and aggregated at the unit level using identification codes to assign records to either HPT or matched control cohorts. Due to the de-identified, unit-level nature of the dataset, individual-level demographic variables (e.g., sex, age, rank) were not available across all outcome domains. Only ACFT and BCP data included sex-disaggregated counts. Therefore, sex was not modeled as a fixed effect. In FY2019, no brigades were HPT-resourced, establishing a clean pre-intervention baseline. A total of 56 brigades were selected on the basis of operational alignment and data completeness, forming two equal cohorts: 28 HPT-resourced brigades and 28 matched controls.

Initial HPT fielding began in FY2020, with most teams reaching minimal operational capacity (~ 30% staffing) by FY2021 and achieving near-full operational capacity staffing levels (> 75%) by FY2022. By FY2023, seven brigades from the original control group had transitioned to HPT resourcing, creating a dynamic exposure structure that required unit-level tracking across all years of analysis. HPT staffing models were tiered on the basis of brigade type and operational need. Tier 1 brigade combat teams, combat aviation, sustainment, and medical brigades received a full complement of 37 personnel; tier 2 field artillery, engineers, air defense artillery, and military police received 25 personnel.; tier 3 basic combat training, one station unit training, and advanced individual training were staffed under a separate model with 29 personnel. While this tiered staffing structure defined expected team composition, actual fill rates and utilization varied by installation and over time. Therefore, treatment was modeled as a binary indicator of HPT presence, rather than as a tiered or fidelity-weighted variable. This approach maintained analytic consistency across years, avoided misclassification, and enabled robust intent-to-treat estimation of average treatment effects across brigade types [75, 79]. Although tiered staffing levels may influence treatment intensity, they were not modeled as random or fixed effects due to small subgroup sizes, potential collinearity with HPT presence, and insufficient statistical power for stratified inference [77]. Preliminary stratified analyses showed directionally consistent treatment effects across tiers, supporting the validity of pooled average estimates and ensuring stability in longitudinal modeling.

### Analytical Framework

This longitudinal program evaluation used a hybrid analytic strategy to address both cross-sectional and temporal questions of effectiveness. Two primary analytic pathways were pursued:

- Q1: *Do HPT-resourced brigades differ significantly from controls in outcome distributions or odds of success?* Estimation of group-based differences in outcomes (HPT versus control), across each fiscal year.
- Q2: *Do outcomes change over time differently in HPT versus control brigades, indicating treatment responsiveness?* Estimation of changes in outcome trajectories over time (i.e., group-by-year trends) to assess treatment effects longitudinally.

All data preprocessing, transformation, and cleaning were performed using Python 3.12 in Jupyter Notebook, employing standard scientific libraries including NumPy, Pandas, SciPy, and Matplotlib. Economic modeling—comprising cost mapping, triangular distribution construction, and Monte Carlo simulation—was implemented using SimPy and NumPy, with triangular sampling validated against reference functions in the random and scipy.stats modules. Post-simulation analyses were performed using both descriptive and inferential statistical methods in Python and JMP Pro (v17.0.0; JMP Statistical Discovery, 2022). Data visualization was conducted in MATLAB (R2024a) and RStudio Pro (R 4.4.1), with the ggplot2 and gridExtra packages used to generate final publication-quality figures. All code was independently validated and archived in a version-controlled reproducibility directory with complete logs and metadata.

#### Analyzing Group-Outcome Relationships (Q1)

To evaluate whether the presence of HPTs significantly altered the distribution of key readiness outcomes, a categorical comparison strategy was used. Group comparisons between HPT and control brigades were conducted for each binary outcome across fiscal years (FY2019–FY2023), using the following analytic sequence:

*Q1a. Is there a statistically significant association between group and outcome?*

Chi-squared tests of independence were used to evaluate whether the distribution of positive and negative outcomes (e.g., ACFT pass/fail, presence/absence of MSKI referral) differed between HPT and control brigades [85, 86].

*Q1b. Which group shows stronger or weaker associations, and where are the differences most concentrated?*

For significant Chi-squared results, post hoc Fisher’s exact tests were used to identify whether a particular group is significantly over- or under-represented in a readiness outcome [87].

*Q1c. What are the odds of a given outcome occurring in one group versus the other?*

Odds ratios (ORs) were calculated to quantify the magnitude and direction of association between group membership and outcome occurrence [88–90].

*Q1d. How have those odds changed over time?*

To detect directional change, ORs were calculated separately for FY2019 (pre-HPT baseline) and FY2023 (post-resourcing) using the same matched cohort structure. These estimates were then compared narratively to assess slope direction and magnitude, enabling intuitive interpretation of responsiveness to HPT resourcing.

*Q1e. What is the projected impact of these differences on force readiness at scale?*

To estimate population-level readiness implications, observed OR-based differences were extrapolated to the active component and total Army force sizes. Proportional changes in event likelihood were used to model: (1) the number of soldiers potentially “spared” from negative events (e.g., injury, attrition); (2) the number newly achieving performance benchmarks (e.g., ACFT pass, expert marksman); and (3) associated resource impacts. These extrapolated event counts served as the foundational inputs for downstream economic modeling.

#### Trend Modeling: Difference-in-Differences (Q2)

A longitudinal change-score method was employed to evaluate how outcomes changed over time and to determine whether HPT resourcing influenced these patterns compared with control brigades. Specifically, this analysis employed a DiD strategy implemented through baseline-adjusted factorial ANOVA. Following recommended guidance for quasi-experimental designs [76–78, 91–98], outcome rates for each unit were normalized to their FY2019 baseline, producing difference scores for each subsequent year (FY2020–FY2023). This approach allows for estimation of treatment effects while controlling for both baseline differences and secular trends.

*Model structure*. A series of mixed factorial ANOVA models were constructed using standard least squares means (LSM) estimation. Each model included:

- Between-subjects factor: H2F resourcing status (HPT versus control)
- Within-subjects factor: fiscal year (four levels: FY2020–FY2023)
- Dependent variable: change in each outcome from FY2019 baseline

Assumptions were verified before modeling, including normality with the Shapiro–Wilk test, homogeneity of variance using Levene’s test, and sphericity assessed with Mauchly’s test. However, ANOVA is generally robust to moderate violations of these assumptions, particularly with large sample sizes and balanced designs [96, 99–102].

*Why DiD ANOVA was chosen over ANCOVA*. Although analysis of covariance (ANCOVA) is a common approach for analyzing pre–post designs, it assumes equal slopes between groups and can obscure true treatment effects when baseline differences are large or group trajectories are non-parallel [91, 92, 103–106]. In contrast, the DiD ANOVA framework: (1) allows for non-parallel trends across groups; (2) models interaction effects (group × year) to isolate treatment impact; and (3) avoids over-adjustment bias that may arise in traditional ANCOVA [94–96]. Moreover, DiD ANOVA accommodates heterogeneity in treatment effects, adjusts for potential compositional shifts over time, and offers more intuitive interpretation of change scores in policy-relevant units (e.g., percent improvement, rate change).

*Q2a. Are there significant group differences in outcome trajectories since resourcing?*

Main effects and interaction terms were examined to determine whether HPT brigades experienced significantly greater (or lesser) changes in outcomes over time. Group-by-year interaction effects were interpreted as evidence of treatment responsiveness.

*Q2b. Which year-to-year comparisons drive observed group differences?*

To specify which differences were statistically significant, all ANOVA models were followed by Tukey’s HSD post hoc tests, adjusted for multiple comparisons [96, 105, 106]. These pairwise tests identified the specific FYs and outcomes where HPT brigades diverged most strongly from controls—helping delineate time-to-effect and peak impact periods for each outcome.

#### Economic Modeling Framework: ROI Estimation Strategy

To translate observed readiness improvements into defensible economic terms for quantifying the short-horizon ROI of embedded HPTs, we developed a multitiered, DiD economic modeling framework. This framework integrates causal effect estimation, deterministic cost attribution, probabilistic simulation, and enterprise-level extrapolation [107–110]. Every layer was created to enable clear, scalable, and cautious financial modeling for Army H2F modernization. Methodological fidelity, paired with outcome-specific modeling, ensures that the results provide not just statistical significance, but also operational and fiscal significance, relevant to Army planners, policymakers, and congressional stakeholders.

- *Observed outcome differences*. Net treatment effects for each outcome were assessed using DiD models. We categorized modeled outcomes into five conditionally exclusive domains: (1) clinical treatment burden, (2) attrition-linked separation risk, (3) remedial performance costs, (4) restored readiness value, and (5) combat inefficiency (i.e., sunk cost of “non-ready” soldiers). These assignments represent short-term Army expenses that can be directly traced to decreases in MSKI-related medical use, the length of BH profiles, immediate separation risks, remedial performance requirements, and the proportion of non-ready forces. No speculative or long-latency benefits (e.g., VA deferral, reenlistment gains) were included. These models adjusted for secular trends and baseline differences, capturing how HPT-resourced brigades diverged from matched control units across time.
- *Cost attribution and unit economics.* Each treatment delta was mapped to a cost-per-case value using conservative, validated benchmarks from four source references: Defense Health Agency (DHA)/TRICARE encounter billing data; Defense Financial Accounting Services (DFAS) composite pay and productivity loss models; Government Accountability Office, RAND, and Defense Business Board (DBB) manpower replacement studies; and peer-reviewed DoD economic evaluations. Triangular distributions were used to estimate costs for each domain, incorporating minimum, most-likely (mode), and maximum values. Domains with higher right-tail risk (e.g., MSKI > 90 days, BH > 90 days, SA profiles) were explicitly modeled with extended maxima, consistent with uncertainty standard best practices [108, 111, 112]. Importantly, the model distinguished between cost avoidance and direct readiness gains. Duty day restoration and expert-level RMQ were considered separate productivity improvements, not avoided losses, and were analyzed alongside the cost domains. These gains were omitted from the cost-avoidance ROI denominator to prevent redundancy; however, they were incorporated into the broader economic assessment to accurately represent measurable advancements in mission-capable time and operational proficiency.
- *Brigade-level cost avoidance estimates*. The change in log odds from FY2019 to FY2023 was calculated individually for both the HPT and control groups for each outcome. The difference between these log odds was calculated to produce a DiD-adjusted treatment effect (ΔlogOR), which was then exponentiated to show the shift in OR ratio caused by HPT resourcing. The absolute risk reduction (ARR) for each domain was estimated using the Zhang and Kai (1998) approximation [113]. The ARRs were then scaled to brigade size (ΔN_d = ARR × 3571) to estimate the number of adverse events avoided annually per brigade. Each domain’s case delta (ΔN_d) was multiplied by a validated “most-likely” cost-per-event (Ĉ_d) to derive gross annual savings. Net cost avoidance was calculated by subtracting a fixed $3.0 million annual implementation cost. This deterministic estimate served as the baseline for Monte Carlo simulation and force-wide extrapolation.
- *Monte Carlo simulation and ROI probability distribution*. A 10,000-draw Monte Carlo simulation was conducted to model uncertainty and estimate probability distributions across plausible ROI thresholds. For simulation, each cost and case delta was sampled from its triangular distribution. Cross-domain dependencies (e.g., comorbid MSKI and BH) were modeled via a Gaussian copula with a fixed interdomain correlation of *ρ* = 0.15, supporting bounded co-occurrence without inflating fiscal impact. This structure introduced bounded, empirically justified uncertainty on both axes—cost and event delta—while preserving the causal integrity of treatment effects. Additionally, we computed a TEV per draw by summing cost avoidance with monetized readiness gains.
- *Force-wide extrapolation to active and total Army*. Force-wide extrapolation was conducted post-simulation to preserve draw-level variance. Using a standardized 3571-soldier denominator, brigade-level savings were scaled to 124 Active-duty (445,000 soldiers) and 266 total Army brigade equivalents (950,000 soldiers). While real-world brigade sizes vary by type, the use of a standardized brigade equivalent enables reproducible scaling and consistent interpretation of per-unit estimates. The extrapolation to Active-duty brigades assumes constant average per-unit effect, acknowledging that real-world variability in leadership, mission tempo, and MOS composition may influence unit-specific outcomes but not invalidate average effects at scale [114, 115]. Extrapolating to the total Army requires consideration of structural and operational differences between the Active and Reserve components, which include the Army Reserve and Army National Guard. Unlike the Active component, Reserve component units are not slated to receive fully embedded HPTs under current modernization plans. Instead, the Reserve component delivery model includes hub-and-spoke regional support centers, mobile teams, and asynchronous subject-matter-expert (SME) access—potentially creating meaningful differences in proximity, frequency, and integration. Reserve component values were discounted by a fidelity factor of 0.65 to reflect differences in SME access and delivery model integration. This fidelity adjustment was applied after Monte Carlo simulation, preserving the draw-level distribution and variance structure. This discount reflects historical differences in health service utilization and readiness intervention fidelity between full-time and part-time units [34, 116–120]. It also aligns with published comparative evaluations of embedded versus remote wellness programs, which consistently show diminished returns under asynchronous or episodic delivery models [13, 52, 121–123]. While this factor is conservative by design, it ensures that extrapolated ROI values account for the unique structural constraints and utilization limitations of the Reserve Component.

This framework conforms to International Society for Pharmacoeconomics and Outcomes Research (ISPOR) and DoD Cost Assessment and Program Evaluation (CAPE) recommendations for bounded, policy-relevant simulation in military economic modeling [108, 112, 124–126], and mirrors prior evaluations of readiness interventions and healthcare modernization efforts in military populations [127, 128]; it also adheres to CAPE and ISPOR best practices [126, 129, 130]. All findings and assumptions were based on sources that can be independently confirmed, and were organized to show the lowest reasonable amount of savings given today’s HPT delivery methods. This conservative framing ensures that ROI estimates can support planning, modernization, and budgeting decisions without overstating benefit magnitude.

#### Cost Attribution Model and Outcome Mapping

To estimate domain-specific cost avoidance, we translated ARRs into monetized readiness gains using outcome-specific unit costs, scaled to the per-brigade level. See Table 1 for the summary triangulation cost for minimum, most-likely (mode), and maximum estimates. All cost estimates reflect a U.S. Army payer perspective unless otherwise specified.

**Table 1:** Triangular cost distributions applied in the economic model

Domain	Outcome	Minimum ($)	Most-likely ($)	Maximum ($)
Clinical treatment and profile burden	MSKI < 90 days	2500	4800	6000
	MSKI > 90 days	9000	12,500	28,000
	MSKI referral	4500	6535	11,000
	BH < 90 days	3600	6700	12,000
	BH > 90 days	9000	14,600	22,000
	SA profile	6000	11,400	18,200
Attrition-linked risk model	SA profile (0.30 risk)	23,400	28,200	39,000
	BH > 90 days (0.30 risk)	23,400	28,200	39,000
	MSKI > 90 days (0.20 risk)	15,600	18,800	26,000
	BCP fail (0.10 risk)	7800	9400	13,000
	ACFT fail (0.10 risk)	7800	9400	13,000
Remedial and performance costs	ACFT fail (remedial)	2400	4000	4800
	BCP fail (remedial)	2000	2700	3500
	RMQ DNQ (remedial)	1200	1500	2000
Readiness gains	Duty day restoration (per day)	205	260	350
	RMQ expert (gain)	400	500	750
Combat burden “performance tax”	Sunk lifecyle replacement ACFT, BCP, or RMQ fail	78,000	94,000	130,000

All values are expressed in constant FY2023 U.S. dollars and parameterized as triangular distributions (minimum, most-likely, maximum) using validated estimates from DFAS, DHA/TRICARE, RAND, DBB, and peer-reviewed sources. Cost estimates reflect only short-horizon, directly attributable Army expenditures. Attrition-linked domains (e.g., MSKI > 90 days, BH > 90 days, SA profiles, BCP failures, ACFT failures) were modeled as sunk-cost-at-risk cases with empirically justified fractional separation risks and full lifecycle replacement costs, including accession, training, and out-processing. Remedial domains (e.g., ACFT fail, BCP fail, RMQ DNQ) capture the short-term cost of retaining and reconditioning underperforming soldiers. Readiness gains (e.g., duty day restoration, RMQ expert) represent monetized productivity restoration and qualification efficiency and were modeled independently to avoid overlap with net-cost domains. Duty days were valued using DFAS FY2023 composite enlisted pay rates

MSKI, musculoskeletal injury; BH, behavioral health; SA, substance abuse; ACFT, Army Combat Fitness Test; BCP, Body Composition Program; RMQ, Rifle Marksmanship Qualification; DNQ, did not qualify

*Clinica1 treatment and profile burden:* This domain includes direct care, private sector treatment, and command-managed monitoring associated with MSKI, BH, and SA profiles. For each profile type, we triangulated cost estimates from Army Public Health Center profile duration tables stratified by ICD-10 category [7, 14], DFAS FY2023 composite enlisted pay and productivity loss models [6, 15, 43], and encounter-level clinical costs from DoD economic studies and technical reports [6, 8, 9, 11, 17, 45–47, 131–140].

For MSKI, modal costs upper bounds extended to $28,000 to reflect chronicity and protracted recovery. BH profile estimates were $6,700 for short duration (mean = 43 days) and $14,600 for long duration (mean = 100 days), with short BH capped at $12,000 to capture cases involving acute crisis stabilization [16, 23, 24, 141, 142].

For SA profiles, modeling emphasized short-term monitoring and treatment burden over confirmed separation. Cost estimates incorporated limited duty productivity loss, weekly command oversight, and standard outpatient behavioral health encounters. On the basis of surveillance data, a most-likely (mode) estimate of $11,400 was used, bounded by a minimum of $6,000 and a maximum of $18,200 to account for cases requiring extended intervention and BH comorbidity [25, 27, 29].

*Attrition-linked risk modeling:* We modeled short-horizon (12–24 month) separation risk from six outcomes with consistent historical correlations to administrative separation. These events were modeled as “sunk-cost-at-risk” cases, but not assumed to culminate in discharge. Instead, we applied conservative conversion factors grounded in published and internal Army sources*:*

- *SA profiles* were assigned a conservative 30% separation probability, reflecting partial progression from monitoring status to administrative discharge. This estimate is supported by longitudinal analyses showing that 35–45% of soldiers in treatment for substance use disorders separate within 24 months [27–29, 143].
- *BH* > *90 days* was discounted to 30%, balancing the known long-term impact of chronic behavioral health issues with internal MHS/Defense Health Board findings showing varied outcomes depending on command support and treatment response [16, 23, 24, 141].
- *MSKI* > *90 days* was modeled with a conservative 20% separation risk. This reflects findings from disability adjudication studies [6, 8–10, 14, 15, 43, 133, 140, 144, 145], indicating that chronic MSKIs are a leading driver of disability discharge, but only a fraction escalate to full attrition within 24 months.
- *BCP failures* were assigned a 10% attrition risk on the basis of internal data and retention studies showing that most flagged soldiers enter remediation pathways and only 5–15% ultimately fail to meet standards after multiple cycles [19–21, 146–149]
- *ACFT failures* were similarly assigned a 10% separation conversion rate. Early evaluations of the ACFT rollout and remedial reconditioning programs show high remediation success rates, though a persistent minority fail to progress [82, 150].

All attrition-linked outcomes were valued using full lifecycle replacement costs derived from validated recruiting, training, and onboarding models [45, 151, 152]. The lifecycle cost per lost soldier (mode = $94,000) was held constant across domains to enable conservative comparability, with upper bounds reaching $130,000 in high-risk cases.

*Remedial and performance costs:* Short-horizon remedial burdens were separately modeled from attrition risk and represent a recurring cost for underperforming but retained soldiers:

- *ACFT failure* (*remedial*) was valued at $4000 (mode) on the basis of reconditioning labor, SME-led remedial PT, retesting logistics, and administrative oversight.
- *BCP Failure* (*remedial*) was valued at $2700 (mode) on the basis of monthly SME-led counseling, retesting, and PT regimens.
- *RMQ DNQ* (*remedial*) used a modal value of $1500, derived from ammunition, range operations, and instructor labor.

*Readiness gains:* Readiness-positive outcomes—including restored duty days and enhanced expert RMQ rates—were modeled as an independent outcome domain to capture short-horizon productivity gains attributable to HPT implementation. These values represent direct readiness restoration rather than cost avoidance and were therefore excluded from the net-cost domains to prevent double counting. Instead, they were reported as a distinct fiscal gain reflecting increased mission-capable time and improved combat effectiveness.

- *Duty day restoration* was modeled as a distinct domain to estimate productivity regained from reductions in limited-duty profiles. Duty day restoration was calculated from DiD-estimated event reductions across five profile types (MSKI < 90 days, MSKI > 90 days, BH < 90 days, BH > 90 days, SA), multiplied by average profile length and DFAS FY2023 pay tables.
- *Expert RMQ* was valued at $500 per added expert, reflecting downstream training savings, improved marksmanship, and increased combat effectiveness.

Together, these readiness-positive outcomes represent short-horizon, directly monetizable improvements in operational availability and performance. Their inclusion supports a more complete accounting of fiscal return by capturing benefits not reflected in traditional cost-avoidance models, while still preserving analytic conservatism through non-overlapping attribution.

*Combat inefficiency burden* (“*performance tax*”). This domain estimates the sunk cost of soldiers who remain in service but are “non-ready” due to failure on core physical readiness or combat skill tasks—ACFT, BCP, or RMQ. These soldiers have already incurred the full spectrum of military investment—recruitment, initial training, occupational qualification, and active-duty pay and benefits—but do not meet the medical or performance criteria required for deployability. Importantly, these individuals: (1) continue to receive full pay and medical benefits; (2) occupy positions intended for fully deployable and combat-effective soldiers; and (3) often require command waivers or restrictions to participate in operational missions. As such, the model treats each non-ready soldier as a sunk-cost liability. Cost attribution is based on full lifecycle replacement value, reflecting the fact that the DoD has already paid to field these individuals but cannot fully realize their intended operational return. This approach provides a conservative, but operationally realistic, estimate of the fiscal burden associated with sustained non-readiness in key physical and combat domains.

### Statistical Assumptions and Sensitivity Analysis

The strength and reliability of this HPT ROI evaluation rest on three foundational pillars: (1) the causal credibility of treatment effect estimates, (2) the conservative and source-validated structure of cost attribution, and (3) the rigor and transparency of the uncertainty modeling. These elements collectively ensure the analytic defensibility of the model and enhance its relevance for Army leaders, health economists, and policy analysts.

*Causal assumptions and treatment effect validity*: The analytic design assumes that in the absence of HPT implementation, the treatment and control brigades would have followed parallel readiness trajectories. Although some baseline imbalance was expected—given that early fielding targeted high-need units—multiple safeguards were incorporated to preserve internal validity. All outcomes were normalized to a shared FY2019 baseline. Group-by-year interaction terms were included to detect non-parallel trends. Post hoc testing confirmed no significant pre-treatment divergence across groups. These design features align with best practices in quasi-experimental program evaluation and support the causal interpretability of the estimated treatment effects despite the absence of random assignment.

*Economic model assumptions and cost attribution*: Each outcome domain was assigned a short-horizon distribution grounded in validated sources. Unit costs were structured as triangular distributions with a conservative minimum, empirically supported mode, and plausible upper bound on the basis of documented cost exposure. No speculative or long-horizon savings—such as disability avoidance, downstream medical deferral, or retention value—were included. This conservative framing ensures that modeled ROI reflects only directly measurable, short-term cost avoidance that can be reasonably attributed to HPT implementation.

*Cross-domain dependencies and correlated risk structure*: To reflect the real-world comorbidity of medical, behavioral, and attrition-linked outcomes in tactical populations, the simulation incorporated a cross-domain dependency structure. This approach preserves the bounded nature of simulation draws while allowing for realistic outcome clustering—e.g., a soldier with a long-duration MSKI profile may also be at risk for BH referral or ACFT failure. This structure aligns with recommendations for multi-domain economic modeling [107–110, 115].

*Simulation credibility and bounded uncertainty*: The 10,000-draw Monte Carlo simulation incorporated bounded uncertainty on both cost and outcome dimensions. For each domain, cost-per-event was sampled from a validated triangular distribution derived from four-source triangulation. Separately, the number of events avoided per brigade was also modeled as a triangular distribution on the basis of the point estimate from DiD and the transformed 95% confidence interval of the log odds ratio. This two-dimensional uncertainty structure preserved the fixed causal estimate per domain while introducing empirically defensible variance consistent with observed fiscal risk profiles.

*Intent-to-treat scope and fidelity considerations:* The analysis used an intent-to-treat (ITT) framework, treating HPT presence as the independent variable of interest [75, 79]. This means that ROI estimates reflect average outcomes under existing conditions—regardless of individual variation in SME utilization, staffing continuity, or command engagement. No attempt was made to stratify brigades by fidelity of implementation or degree of SME integration. Therefore, the reported ROI figures represent cautious, real-world estimates on the basis of current HPT delivery practices. They probably underestimate the highest possible benefit of fully integrated, high-fidelity delivery models.

## Results

*Participants*: In fiscal year (FY) 2019, the baseline cohort comprised 201,727 soldiers across 56 brigades (mean per brigade = 3602 ± 1820 SD; mean age = 27.11 ± 1.34 years SD). By FY2020, 101,123 soldiers were assigned to HPT-resourced brigades, while 98,180 remained in control units without embedded teams. Group sizes and ages remained comparable in FY2021 (HPT: *n* = 106,612; control: *n* = 103,676) and slightly decreased in FY2022 (HPT: *n* = 105,766; control: *n* = 102,771). By FY2023, seven brigades transitioned from control to HPT status, yielding 133,484 soldiers in the HPT cohort (brigade mean *n* = 3813 ± 1739; age = 27.94 ± 1.23) and 67,703 soldiers in the control group (*n* = 3223 ± 1607; age = 28.33 ± 1.32).

*Outcome trajectories and effect modeling*: The results of group-by-year interaction effects, tested using full factorial least-squares mean (LSM) ANOVA models across all 5 years of the evaluation (FY2019–FY2023), are presented in Table 2.

**Table 2:** Significant changes in outcome rates over time: group-by-FY interactions

HPT ROI metric	*df*	SS	MS	*F* ratio	*p*	*R*^2^
MSKI profile	8	2290.63	286.33	14.007	< 0.0001	0.2925
MSKI profile > 90 days	8	1392.57	174.07	8.049	< 0.0001	0.1920
MSKI referral	8	680.69	85.09	7.058	< 0.0001	0.1724
BH profile	8	50.67	6.33	5.871	< 0.0001	0.1477
BH profile > 90 days	8	24,683.04	3085.38	6.617	< 0.0001	0.1634
SA profile	8	51.79	6.47	8.729	< 0.0001	0.2049
Fail BCP	8	841.49	105.19	36.343	< 0.0001	0.5176
Pass ACFT	8	77,657.43	9707.18	6.518	< 0.0001	0.1614
RMQ expert	8	6711.09	838.89	6.200	< 0.0001	0.1547

This table presents the results of full factorial LSM ANOVA models evaluating group-by-year interaction effects across FY2019–FY2023. For each outcome metric, HPT and control brigades exhibited significantly different slopes over time

d.f., degrees of freedom; SS, sum of squares; MS, mean square; MSKI, musculoskeletal injury; BH, behavioral health; SA, substance abuse; BCP, Body Composition Program; ACFT, Army Combat Fitness Test; RMQ, Rifle Marksmanship Qualification

### Readiness for Duty

In military operations, readiness is defined by the Department of Defense as “the ability of military forces to fight and meet the demands of assigned missions” [153]. At the individual level, medical readiness includes the absence of injury or illness that could impair occupational performance or deployability. In this analysis, we focus on early indicators of non-deployability, including long-duration MSKI profiles, BH conditions, and active SA diagnoses.

We find that:

- Prior to HPT resourcing, brigades designated to receive teams exhibited higher baseline rates of all adverse readiness outcomes.
- Following implementation, these brigades showed significant improvements—eventually reversing the odds of negative outcomes compared with controls.

**Fig. 1:**
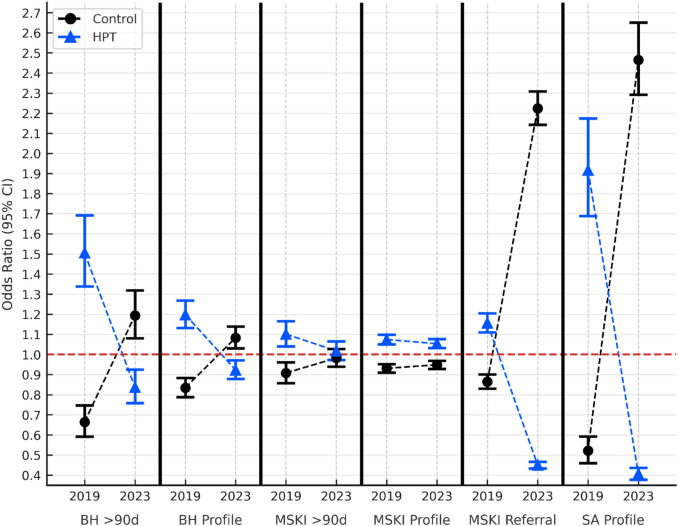
Change in readiness for duty odds ratios since H2F performance team resourcing. Odds ratios (ORs) and 95% confidence intervals (CIs) for H2F Performance Team (HPT) brigades (blue triangles) versus control brigades (black circles) are shown for FY2019 (pre-fielding) and FY2023 across key readiness for duty outcomes. The horizontal dotted line at OR 1.0 denotes no difference between groups. Values above this line indicate higher odds of the outcome occurring; values below indicate lower odds. None of the outcomes fall on the line—demonstrating significant between-group differences at all timepoints. This figure shows how HPT-resourced units, which started with significantly worse readiness indicators (e.g., OR 1.9162 for SA profiles in FY2019), reversed trends by FY2023 (e.g., OR 0.4056), while control units often worsened over time. BH, behavioral health; MSKI, musculoskeletal injury; SA, substance abuse

Longitudinal Chi-squared differences in readiness for duty probability are described below (and summarized in Supplementary Table 2 and Supplementary Table 3, Online Resources 1). The group-based change in outcome odds ratios from FY2019 to FY2023 are illustrated in Fig. 1.

#### Musculoskeletal Injury (MSKI) Profiles

*Trend analysis.* A significant group-by-year interaction was observed for MSKI profile rates (*F*(8) = 14.007, *p* < 0.0001). While both groups experienced rising rates over time, the increase was substantially larger in control brigades. Post hoc Tukey HSD comparisons revealed year-over-year spikes in FY2021 (*Δ* = 3.37; SE 0.85; *p* = 0.0001) and FY2023 (*Δ* = 4.90; SE 0.87; *p* < 0.0001), with FY2023 showing the most pronounced divergence from FY2019 (*Δ* = 7.26; SE 1.06; *p* < 0.0001). Control brigades exhibited a steeper increase (*Δ* = 7.78; SE 1.16) than HPT units (*Δ* = 6.74; SE 1.55).

*Probability and odds:* Prior to HPT resourcing, future HPT brigades had significantly higher odds of MSKI profiles (OR 1.0741; 95% CI 1.0501–1.0987). By FY2023, this gap narrowed slightly (OR 1.0539; 95% CI 1.0317–1.0765) but remained statistically significant. Fisher’s exact tests confirmed consistently elevated profile probabilities in HPT brigades until FY2023, when the trend began to stabilize.

MSKI Profiles > 90 Days.

*Trend analysis:* A significant group-by-year interaction was observed for long-duration MSKI profiles (*F*(8) = 8.049, *p* < 0.0001), with a marked spike in FY2023. Tukey HSD post hoc comparisons confirmed this trend, showing that control brigades experienced a significantly greater increase from FY2019 to FY2023 (*Δ* = 7.89; SE 1.90; 95% CI 4.09–11.69; *p* < 0.0001) than HPT brigades (*Δ* = 5.79; SE 1.00; 95% CI 3.85–9.83; *p* < 0.0001).

*Probability and odds:* Prior to fielding, HPT-bound brigades had significantly higher odds of MSKI profiles exceeding 90 days (OR 1.1011; 95% CI 1.0404–1.1653). By FY2023, this disparity fully disappeared (OR 1.0180; 95% CI 0.9732–1.0649), indicating statistical equivalence across groups.

#### MSKI Referrals to Civilian Care

*Trend analysis:* Referral rates to external civilian providers showed the most pronounced divergence across all MSKI-related outcomes. Control brigades exhibited a significant increase from FY2019 to FY2023 (*Δ* = 5.29; SE 0.89; 95% CI 2.46–8.12;* p* < 0.0001), whereas HPT brigades showed no significant change in referral rates over any year-to-year comparison. By FY2023, the control group’s increase remained significantly greater than that of the HPT group (*Δ* = 3.71; SE 0.96; 95% CI 0.65–6.77; *p* = 0.0052).

*Probability and odds:* Initially, brigades scheduled to receive HPTs were more likely to issue civilian MSKI referrals (OR 1.1565). However, by FY2023, the pattern reversed: HPT brigades were less than half as likely to refer out (OR 0.4496; 95% CI 0.4331–0.4668), while control brigades became over twice as likely (OR 2.2240; 95% CI 2.1424–2.3087). Yearly comparisons (all *p* < 0.0001) confirmed this shift as one of the most dramatic reversals in the dataset. The effect was particularly stark from FY2022 (*χ*^2^ = 22.58) to FY2023 (*χ*^2^ = 1785.68).

#### Behavioral Health (BH) Profiles

*Trend analysis:* A significant group-by-year interaction was detected for BH profiles (*F*(8) = 5.871, *p* < 0.0001). Control brigades were the only group to experience a statistically significant increase over time, with a net change from FY2019 to FY2023 of *Δ* = 1.23 (SE 0.27; 95% CI 0.38–2.08; *p* = 0.0002). In contrast, HPT-resourced brigades maintained stable BH profile rates across all years.

*Probability and odds:* Prior to HPT implementation, treated brigades exhibited significantly higher odds of BH profiles (OR 1.1981; 95% CI 1.1316–1.2686). By FY2023, this pattern had reversed. Control units showed higher risk (OR 1.0833; 95% CI 1.0306–1.1387), while HPT brigades showed a protective trend (OR 0.9231; 95% CI 0.8782–0.9703).

#### BH Profiles > 90 Days

*Trend analysis:* Long-duration BH profiles (> 90 days) showed marked divergence between groups. Control brigades experienced a significant post-FY2019 increase (*Δ* = 34.07; SE 5.53; 95% CI 16.44–51.70; *p* < 0.0001), while HPT brigades remained statistically stable across all time points.

*Probability and odds:* At baseline (FY2019), brigades later assigned HPTs exhibited significantly higher odds of prolonged BH profiles (OR 1.5051; 95% CI 1.3386–1.6923). By FY2023, the relationship had fully reversed: control brigades had increased odds (OR 1.1943; 95% CI 1.0812–1.3192), while HPT brigades had significantly reduced odds (OR 0.8373; 95% CI 0.7580–0.9249).

#### Substance Abuse (SA) Profiles

*Trend analysis:* SA profiles increased significantly over time in control brigades (*Δ* = 1.53; SE 0.22; 95% CI 0.83–2.23; *p* < 0.0001), while HPT brigades maintained stable levels throughout the study period. Importantly, the 95% confidence intervals for SA profile rates narrowed significantly in HPT brigades while widening in control units—highlighting not only the mean risk reduction, but also increased predictability and control in BH-related readiness risks under embedded support.

*Probability and odds:* Before resourcing, future HPT units were nearly twice as likely to generate SA profiles (OR 1.9162; 95% CI 1.6886–2.1744). By FY2023, the odds had shifted dramatically. HPT brigades showed significantly lower risk (OR 0.4056; 95% CI 0.3772–0.4362), while control brigades exhibited more than 2.4 times the odds of SA profile occurrence (OR 2.4653; 95% CI 2.2924–2.6511). The magnitude of difference increased substantially from FY2022 (*χ*^2^ = 15.12) to FY2023 (*χ*^2^ = 631.12), making this one of the strongest risk reversals observed across all domains.

### Combat Performance Readiness

**Fig. 2:**
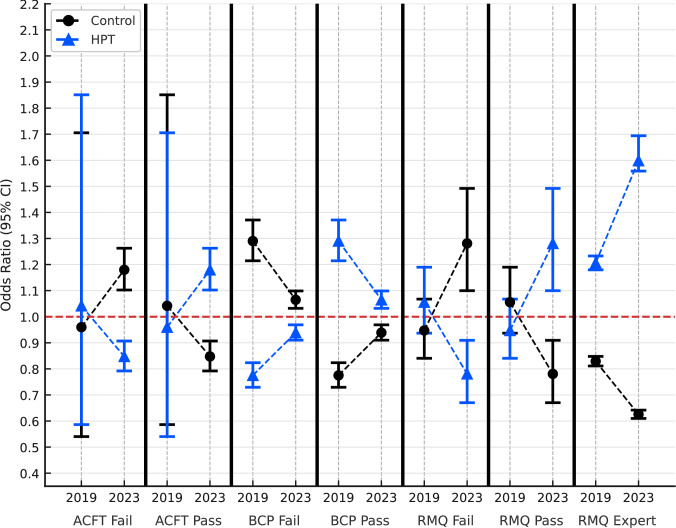
Change in performance readiness odds ratios since H2F performance team resourcing. Odds ratios (ORs) and 95% confidence intervals (CIs) for H2F Performance Team (HPT) brigades (blue triangles) versus control brigades (black circles) are shown for FY2019 and FY2023 across performance readiness outcomes. The horizontal dotted line at OR 1.0 represents no difference between groups. Outcomes below the line indicate reduced odds; outcomes above the line indicate elevated odds. In FY2019, ACFT and RM qualification rates did not differ significantly (OR ≈ 1.0), but all other measures showed significant between-group differences. By FY2023, HPT brigades had significantly higher odds of favorable performance outcomes, such as expert rifle marksmanship (e.g., OR 1.5985), while control units were significantly less likely (e.g., OR 0.6256). ACFT, Army Combat Fitness Test; BCP Army Body Composition Program

Combat performance readiness indicators reflect critical physical and motor capabilities essential for lethality and survivability in operational environments. These measures include compliance with Army body composition standards, physical fitness testing, and marksmanship performance—each independently associated with combat effectiveness, injury risk, and retention [154, 155]. The statistical trajectory of outcomes is described below using both group-level longitudinal analysis and within-year comparisons. The group-based change in outcome odds ratios from FY2019 to FY2023 are shown in Fig. 2. Supplementary Table 4 (Online Resources 1) summarizes the longitudinal Chi-squared differences in performance readiness probability between control and HPT resourced cohorts across all FYs.

#### Army Body Composition Program (BCP) Failure

*Trend analysis:* BCP failure rates increased significantly across both groups over the study period (*F*(8) = 36.343, *p* < 0.0001), with the largest spike occurring in FY2023. Although the group-by-year interaction was not significant, the absolute increase in FY2023 was substantial (*Δ* = 4.39; SE 0.40; 95% CI 3.30–5.49). Confidence intervals for BCP outcomes also narrowed modestly across both groups by FY2023, suggesting improved consistency in body composition compliance monitoring and intervention delivery.

*Probability and odds:* Despite similar trajectories, HPT brigades consistently exhibited lower odds of BCP failure across all years (*p* < 0.0001). In FY2019, control brigades were 29% more likely to fail (OR 1.2903; 95% CI 1.2144–1.3708). Although this gap narrowed slightly, the difference remained significant in FY2023 (OR 1.0649; 95% CI 1.0321–1.0988).

#### Army Combat Fitness Test (ACFT)

*Trend analysis:* ACFT pass rates improved significantly in both groups from FY2019 to FY2023 (*F*(8) = 6.518, *p* < 0.0001), but HPT brigades showed a larger gain. The net increase for HPT brigades was *Δ* = 48.89 points (95% CI 6.63–91.16; *p* = 0.0100), compared with *Δ* = 39.00 for control brigades (95% CI 7.50–70.50; *p* = 0.0039), indicating superior physical readiness acceleration under HPT support. Confidence intervals also narrowed substantially by FY2023 in both groups, indicating reduced interindividual variance and increased precision in pass rate estimates.

*Probability and odds:* Pre-fielding, the two groups had statistically equivalent ACFT outcomes (*p* = 0.8889). Starting in FY2020, however, HPT brigades demonstrated significantly higher pass rates in every subsequent year (*p* < 0.0001). By FY2023, control brigades were 17.98% more likely to fail (OR 1.1798; 95% CI 1.1025–1.2626), while HPT brigades were significantly less likely to fail (OR 0.8476; 95% CI 0.7920–0.9070). Paired *t*-tests further confirmed superior average scores among HPT soldiers (mean *Δ* = 12 points; 95% CI 503.24–503.58; *p* = 0.0353).

#### Rifle Marksmanship Qualification

*Trend Analysis:* No significant group-by-year interaction was found for basic rifle marksmanship outcomes (*F*(8) = 1.522, *p* = 0.1492), likely due to across-the-board improvements in the force and changes to RMQ procedures in FY2021. However, performance trends remained directionally favorable for HPT brigades.

*Probability and odds:* HPT brigades outperformed control units in three of the four post-resourcing years (FY2020, FY2022, FY2023). In FY2023, the group difference was statistically significant (*p* = 0.0010), with control units 28% more likely to fail (OR 1.2810; 95% CI 1.0997–1.4921) and HPT brigades significantly less likely to fail (OR 0.7807; 95% CI 0.6702–0.9093).

#### Expert Rifle Marksmanship Qualification

*Trend analysis:* Expert-level qualification (≥ 90% accuracy) showed the most pronounced effects among performance metrics. A significant group-by-year interaction was observed (*F*(8) = 6.200, *p* < 0.0001). Control brigades experienced a sharp decline in FY2023 (*Δ* =  − 11.43; 95% CI − 1.93 to − 20.92; *p* = 0.0059), whereas HPT brigades maintained stable expert qualification rates throughout.

*Probability and odds:* In every post-resourcing year, HPT brigades had significantly higher odds of expert qualification (*p* < 0.0001). By FY2023, expert qualification rates reached nearly 44% in HPT units, compared with just under 34% in control brigades (*p* = 0.0265; 95% CI 38.70–39.03). The odds ratio increased from 1.2060 pre-fielding (FY2019) to 1.5985 by FY2023 in HPT brigades, while declining to 0.6256 in control brigades—a 60% relative improvement attributed to HPT support.

### Economic Return on Investment

Building on these readiness and performance findings, we examined the fiscal implications of embedded HPTs. Using the DiD-estimated event reductions and the cost-attribution framework, we translated changes in programmatic outcomes into annual per-brigade cost avoidance and readiness gains. We first present deterministic, outcome-specific estimates, then summarize the results of a 10,000-draw Monte Carlo simulation to generate probabilistic estimates of TEV and ROI at both the brigade and force levels.

#### Outcome-by-Outcome Cost Avoidance Estimates

Using multiyear DiD-estimated event reductions, we calculated deterministic, per-brigade annual cost avoidance for each outcome domain (see Table 3). Brigade-level cost avoidance was primarily concentrated in MSKI-related outcomes, which collectively yielded more than $5.2 million annually in direct avoidance per brigade.

**Table 3:** Annual deterministic cost by outcome domain (FY2023 USD)

Domain	Outcome	Events avoided per brigade	Brigade-level savings ($)	Active-duty savings ($M)
Clinical treatment and profile burden	MSKI < 90 days	261	1,252,800	155.35
	MSKI > 90 days	180	2,250,000	279.00
	MSKI referral	266	1,783,310	215.55
	BH < 90 days	27	180,900	22.43
	BH > 90 days	32	467,200	57.93
	SA profile	18	205,200	25.45
	Subtotal: profile burden	784	6,139,410	755.71
Attrition-linked risk model	SA profile (0.30 risk)	18	507,600	62.54
	BH > 90 days (0.30 risk)	32	902,400	111.9
	MSKI > 90 days (0.20 risk)	180	3,384,000	419.4
	BCP fail (0.10 risk)	12	112,800	13.99
	ACFT fail (0.10 risk)	120	1,128,000	139.8
	Subtotal: attrition risk	362	6,034,800	747.63
Remedial and performance costs	ACFT fail (remedial)	120	480,000	59.53
	BCP fail (remedial)	12	32,400	4.02
	RMQ DNQ (remedial)	85	127,500	15.81
	Subtotal: remediation	312	639,900	79.36
Total cost avoidance	–	1363	12,814,110	1,583
Readiness gains	Duty day restoration (/day)	37,484 days	9,746,000	1,208
	RMQ expert (gain)	358	179,000	22.20
	Subtotal: readiness gains	–	9,925,000	1,230.70
Total economic value	–	–	22,739,110	2.81 billion
Combat burden “performance tax”	Sunk lifecyle replacement ACFT, BCP, or RMQ fail	217	20,398,000	2529
Total (all domains)	–	–	43,137,110	5.34 billion

All values reflect per-brigade deterministic point estimates on the basis of DiD-adjusted event reductions and most-likely unit costs (FY2023 USD). Each outcome was assigned to one of five mutually exclusive domains: (1) clinical treatment, (2) attrition-linked separation risk, (3) remediation, (4) readiness gains, or (5) combat inefficiency. Clinical costs were applied to all limited-duty profiles except for the fraction modeled as separating, which was captured under attrition. Remedial costs applied only to underperforming soldiers retained and reconditioned. Readiness gains—including duty day restoration and RMQ expert—were monetized but treated as value accruals rather than cost avoidance. Combat burden reflects retained but non-deployable soldiers who required lifecycle replacement to restore mission-capable force structure. Profile durations for duty day restoration were sourced from APHC surveillance data and peer-reviewed readiness studies: 37 days (MSKI < 90 days), 123 days (MSKI > 90 days), 43 days (BH < 90 days), 100 days (BH > 90 days), and 75 days (SA)

Beyond cost avoidance, HPT brigades gained an additional $9.9 million per brigade in restored productivity every year. This includes 37,484 duty days restored and 358 additional RMQ Expert qualifications, representing monetized operational value but excluded from net-cost domains to prevent double counting. Finally, sustained non-readiness among soldiers failing core physical or marksmanship standards imposed an estimated $20.4 million in latent fiscal burden.

#### Monte Carlo Simulation Uncertainty Modeling: Cost Avoidance, Readiness Gains, and Total Economic Value

**Fig. 3:**
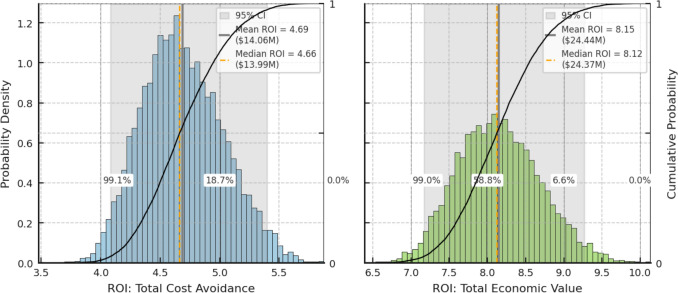
Distribution of brigade-level ROI estimates from 10,000-draw Monte Carlo simulation. This figure shows the simulated distribution of annual per-brigade ROI values generated from a 10,000-draw Monte Carlo analysis evaluating the fiscal return of embedded HPTs. Light blue (total cost avoidance) and green bars (total economic value) represent the empirical histogram of ROI values; the black curve indicates the cumulative probability of achieving at least each level of ROI. The solid vertical line marks the mean ROI, and the dashed orange line marks the median. The shaded gray region represents the 95% confidence interval (CI). Vertical dashed lines mark benchmark ROI thresholds, with adjacent annotations showing the percentage of draws exceeding each benchmark

The Monte Carlo simulation produced a robust, stable, slightly right-skewed distribution of fiscal returns across 10,000 draws. All draws exceeded the break-even threshold, and the mean annual cost avoidance approached $14.1 million per brigade, exclusive of additional readiness gains. The final simulation outputs are presented in Table 4 and shown in Fig. 3.

**Table 4:** Core simulation results for annual economic value (per-brigade, 10,000 draws)

Metric	Total cost avoidance	Readiness gains	Total economic value
Mean	$14.06 million	$10.38 million	$24.44 million
Median	$13.99 million	$10.28 million	$24.37 million
Standard deviation (SD)	$1.02 million	$1.27 million	$1.62 million
95% confidence interval	$12.25 M–16.19 million	$8.15 M–13.00 million	$21.52 M–27.80 million
Minimum observed value	$10.43 million	$7.16 M	$19.18 million
Maximum observed value	$17.64 million	$14.64 M	$30.41 million
Mean ROI/gain/TEV	4.69:1	3.46:1	8.15:1
95% CI (ROI/gain/TEV)	4.08–5.40	2.71–4.33	7.17–9.27
Draws > breakeven (1.0)	100%	100%	100%
Draws > $10 million gross	100%	58.64%	100%

This table presents the core results from a 10,000-draw Monte Carlo simulation estimating the short-horizon economic value of embedded HPTs per active-duty brigade. The simulation incorporated bounded triangular distributions for both cost and case count inputs, with interdomain correlation (*ρ* = 0.15). “Total cost avoidance” reflects modeled reductions in MSKI, BH, attrition-linked, and remedial cost domains. “Readiness gains” quantify monetized productivity recovery from restored duty days and enhanced rifle marksmanship qualification. “Total economic value” represents the combined fiscal effect of both avoided costs and gained readiness, while ROI values are net of a fixed $3.0 million implementation cost

*Threshold probabilities:* To support policy-relevant interpretation of the simulation results, we computed the percentage of draws exceeding key ROI benchmarks. These threshold probabilities (Table 5) quantify the strength and reliability of the fiscal return signal across the full range of simulated outcomes.

**Table 5:** Threshold exceedance probabilities (Monte Carlo, 10,000 draws)

ROI threshold	Cost avoidance ROI (%)	Readiness gains ROI (%)
ROI > 1.0 (breakeven)	100	100
ROI > 2.0	100	100
ROI > 3.0	100	85.77
ROI > 4.0	99.05	11.40
ROI > 5.0	18.73	0.00

This table presents the percentage of simulation draws exceeding key ROI thresholds for both cost avoidance and readiness gains on the basis of a 10,000-draw Monte Carlo simulation. Cost and case inputs were sampled from triangular distributions with interdomain correlation (*ρ* = 0.15), reflecting real-world variability and plausible overlap among outcome domains. Threshold exceedance probabilities quantify the likelihood of achieving specified ROI levels, providing a decision-relevant signal of the fiscal reliability of embedded HPT implementation under conservative modeling assumptions

#### Highest-ROI Domains and Fiscal Contribution Breakdown

**Fig. 4:**
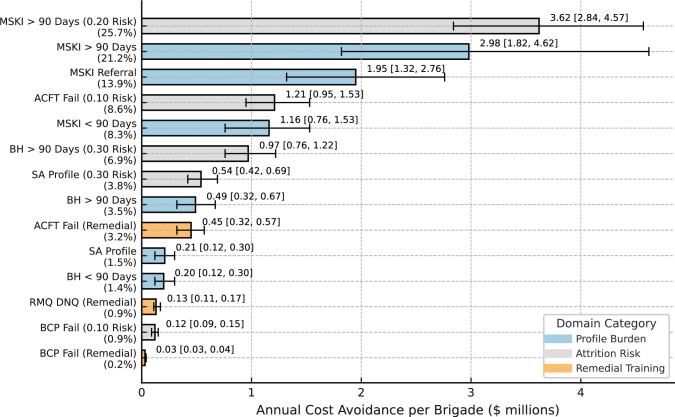
Monte Carlo contribution of outcome to overall annual cost avoidance from embedded HPTs. This tornado plot presents the modeled per-brigade cost avoidance (in millions of FY2023 USD) across 14 outcome domains, ranked top to bottom by mean return. Horizontal bars show the mean cost avoidance per domain, while whiskers represent the 95% confidence intervals derived from 10,000-draw Monte Carlo simulation using triangular distributions for cost inputs and Gaussian copula modeling of interdomain correlation (*ρ* = 0.15). The percentage listed beneath each domain name on the *y*-axis reflects its share of the total return on investment (ROI). Numeric labels adjacent to each bar denote the mean and 95% CI for that domain’s fiscal contribution

To identify the primary fiscal drivers of HPT-related savings, we ranked all modeled outcome domains by their mean per-brigade cost avoidance values derived from the Monte Carlo simulation (see Table 6 and Fig. 4).

**Table 6:** Annual economic contributions by ROI domain (per-brigade Monte Carlo simulation mean, FY2023 USD)

Domain	Outcome	Mean ($M)	SD ($M)	95% CI ($M)
Clinical treatment and profile burden	MSKI < 90 days	1.16	0.2	0.76–1.53
	MSKI > 90 days	2.98	0.77	1.82–4.62
	MSKI referral	1.95	0.38	1.32–2.76
	BH < 90 days	0.20	0.05	0.12–0.30
	BH > 90 days	0.49	0.09	0.32–0.67
	SA profile	0.21	0.05	0.12–0.30
	Subtotal: profile burden	6.99	0.89	5.47–8.88
Attrition-linked risk model	SA profile (0.30 risk)	0.54	0.07	0.42–0.69
	BH > 90 days (0.30 risk)	0.97	0.12	0.76–1.22
	MSKI > 90 days (0.20 risk)	3.62	0.45	2.84–4.57
	BCP fail (0.10 risk)	0.12	0.02	0.09–0.15
	ACFT fail (0.10 risk)	1.21	0.15	0.95–1.53
	Subtotal: attrition risk	6.46	0.49	5.59–7.48
Remedial and performance costs	ACFT fail (remedial)	0.45	0.07	0.32–0.57
	BCP fail (remedial)	0.03	0.00	0.03–0.04
	RMQ DNQ (remedial)	0.13	0.02	0.11–0.17
	Subtotal: remediation	0.61	0.07	0.48–0.74
Total cost avoidance	–	14.06	1.02	12.25–16.19
Readiness gains	Duty day restoration (/day)	10.18	1.27	7.94–12.80
	RMQ expert (gain)	0.20	0.03	0.15–0.26
	Subtotal: readiness gains	10.38	1.27	8.14–13.00
Total economic value	–	24.44	1.62	21.52–27.80
Combat burden “performance tax”	Sunk lifecyle replacement ACFT, BCP, or RMQ fail	21.80	2.71	17.20–27.62
Total (all domains)	–	46.24	3.17	40.52–52.89

This table presents the modeled economic contribution of each outcome domain to per-brigade return on investment (ROI) on the basis of 10,000-draw Monte Carlo simulation outputs. Estimates reflect the mean, standard deviation (SD), and 95% confidence interval (CI) of annual cost avoidance or readiness gains per HPT-resourced brigade in FY2023 USD. Unit costs were modeled as triangular distributions, and interdomain correlation was included (*ρ* = 0.15) using a Gaussian copula. Readiness gains (duty day restoration and RMQ expert) are shown separately from cost avoidance to preserve modeling transparency. The “performance tax” represents the lifecycle replacement burden of soldiers failing core readiness standards and is excluded from the total economic value to maintain a conservative ROI estimate

Extrapolated Cost Avoidance and Net ROI Across Army Force Structures.

Unlike Table 6, which reflects point estimation, force-level extrapolation (presented in Table 7) is based on the Monte Carlo simulation mean, incorporating draw-level variability from triangular cost and case distributions calibrated per domain.

**Table 7:** Force-level extrapolation of HPT annual economic value (FY2023 USD)

Component	Gross cost avoidance	Readiness gains	Implementation cost	Net cost avoidance	Total economic value
Per brigade	$14.06 million	$10.38 million	$3.0 million	$11.06 million	$24.44 million
Active duty (124)	$1.743 billion	$1.287 billion	$372 million	$1.371 billion	$3.030 billion
Reserve (142@0.65)	$1.296 billion	$956 million	$277 million	$1.019 billion	$2.252 billion
Total army (266)	$3.039 billion	$2.243 billion	$649 million	$2.390 billion	$5.282 billion

This table presents annual, per-brigade, and scaled force-wide estimates derived from the 10,000-draw Monte Carlo simulation. Cost avoidance includes reductions in MSKI, BH, attrition-linked, and remedial domains. Readiness gains reflect monetized recovery from duty day restoration and expert RMQ qualification. Total economic value (TEV) combines both cost avoidance and readiness gains. Reserve component estimates apply a conservative 0.65 fidelity discount. All figures are indexed to FY2023 US dollars and reflect only short-horizon, directly attributable Army expenditures; downstream or speculative benefits are excluded

To quantify the fiscal efficiency of embedded HPTs, we calculated the cost-effectiveness ratio (CER) for each modeled category:

- Cost avoidance CER = $14.06 million/$3.0 million = 4.69:1 (95% CI 4.08–5.40)
- Readiness gain CER = $10.38 million/$3.0 million = 3.46:1 (95% CI 2.71–4.33)
- Total economic value CER = $24.44 million/$3.0 million = 8.15:1 (95% CI 7.17–9.27)

This means that for every $1 invested in HPTs, the Army avoided $4.69 in costs and gained $3.46 in readiness improvements, for a combined $8.15 in fiscal value. These ratios were derived from the Monte Carlo simulation’s mean output, reflecting expected, short-horizon return under real-world cost variability. Long-latency or speculative benefits—such as disability deferral, health system offsets, or retention effects—were excluded, underscoring the conservative, policy-relevant nature of these estimates.

## Discussion

This study is the first large-scale evaluation of a holistic human performance optimization model across operational Army units (e.g., H2F). It provides the most robust, real-world and simulation-calibrated evidence to date that embedded HPTs materially improve readiness, combat fitness, and short-horizon fiscal performance. These results are not abstract—they are grounded in multiyear DiD estimates of real-world outcomes, scaled using validated DoD cost benchmarks and conservative structural assumptions.

### Summary of Findings

Across 56 matched brigades and over 1,000,000 soldiers, HPT resourcing reversed adverse trends in injury, behavioral health, and performance outcomes. Despite starting with significantly worse readiness indicators, HPT brigades outperformed controls by FY2023 in nearly every domain. MSKI referrals were reduced by ∼61% (OR 0.4496), reversing a 138 percentage-point disadvantage from FY2019 to a net advantage by FY2023. SA profile risk declined by ∼79% (OR 0.4056), representing a 239 percentage-point reversal from more than 90% higher odds to less than half the risk of controls. BH > 90 days profiles declined by ∼44% (OR 0.8373), flipping from a 50% elevated risk to a 16% protective effect—an effective 66 percentage-point swing. ACFT failure odds dropped by ∼22% (OR 0.8191), reversing from approximate parity in FY2019 to a 21-point advantage. RMQ Expert qualification odds increased by ∼33% (OR 1.5985), moving from a ∼20% advantage to ∼60% higher odds—a 40-point performance gain.

Cost modeling based on DiD-adjusted event reductions and verified unit cost inputs showed that these outcome gains translated into an annual average $14.06 million in cost avoidance and $10.38 million in readiness value per brigade, yielding a total economic value of $24.44 million and a mean ROI of 8.15:1 (95% CI 7.17–9.27). The ROI distribution was highly stable across 10,000 Monte Carlo draws, with 100% of draws above breakeven and 99.05% exceeding 4:1. Importantly, these estimates exclude the $20.4 million per brigade “performance tax” associated with retained but “non-ready” soldiers—an unmodeled fiscal burden that, while omitted from TEV for conservatism, underscores the urgent need for preventive performance support. Force-wide extrapolation yields $5.282 billion in annual total Army savings under conservative assumptions.

#### Cost Avoidance and Operational Efficiency

The economic impact of embedded HPTs is both statistically robust and operationally decisive. On the basis of DiD-estimated event reductions and conservative cost inputs, HPTs generated an average annual cost avoidance of $14.06 million per brigade (95% CI $12.25–16.19 million), yielding a CER of 4.69:1 (95% CI 4.08–5.40). In 10,000-draw Monte Carlo simulations, 100% of ROI estimates exceeded both breakeven and $10 million in annual savings, and 99.05% surpassed a 4:1 return—demonstrating strong fiscal reliability under uncertainty.

Top savings drivers included MSKI referrals ($1.95 million), long-duration MSKI profiles ($2.98 million), and MSKI-related attrition risk ($3.62 million). Additional savings were realized through reductions in BH and SA profiles—especially those exceeding 90 days. Each of these outcomes corresponds to high-cost readiness degraders long documented in Army economic models [6, 8–10, 15, 16, 23, 25, 27, 29, 45, 133, 141]. Notably, out-of-network MSKI referrals routinely exceed $6000–11,000 per episode. Chronic MSKI and BH profiles may cost $10,000 + per soldier, considering imaging, case management, and lost readiness. HPTs disrupted these trajectories through early intervention, embedded care, and load regulation.

These savings exclude long-latency benefits such as disability deferral, reenlistment, or VA spillover reduction. They also omit the “performance tax” of $20.4 million per brigade annually: the sunk cost of retaining soldiers who fail deployability standards. Though excluded from TEV to maintain modeling conservatism, this hidden burden reinforces the strategic need for upstream readiness interventions. These findings reinforce the reliability, scalability, and strategic value of embedded HPTs as a force-wide investment in high-yield, readiness infrastructure.

#### Enhanced Readiness for Duty

Despite worse baseline risk, HPT brigades outperformed controls across all medical readiness indicators by FY2023—including MSKI referrals, profiles, BH outcomes, and SA events. These gains restored deployable strength and accelerated return to duty.

*MSKI referrals and profiles*: HPT brigades achieved a 138 percentage-point reversal in referral odds—dropping from 15.6% higher odds in FY2019 to 55% lower odds by FY2023 (OR 1.1565 to 0.4496). Embedded physical and occupational therapists, athletic trainers, and strength coaches enabled rapid evaluation and prehabilitation, reducing both short- and long-duration MSKI profiles.

*BH and SA profiles*: BH indicators followed a similar trajectory. HPT brigades reversed a 50% disadvantage in BH > 90 days profiles into a 16% advantage (OR 0.8373)—a 66-point swing. SA profile odds dropped from 1.92 to 0.41 (OR), a 239-point reversal. These results reflect the impact of on-site mental readiness personnel, BH educators, and chaplains delivering early intervention through trust-based access. In addition to reducing mean SA profile rates, HPT brigades also exhibited marked tightening of 95% confidence intervals, indicating greater outcome consistency and reduced volatility in BH-related risk. In contrast, control brigades experienced widening CIs over time, suggesting a growing unpredictability in SA outcomes in the absence of embedded support. This stabilization effect reflects not only improved readiness averages, but also enhanced reliability in BH risk mitigation.

*Duty day restoration*: Profile reductions translated to 37,484 duty days restored annually per brigade, valued at $9.7 million. Though excluded from the cost avoidance total, this directly reflects increased force availability and validates HPTs as force sustainment assets.

#### Strengthened Combat Performance Readiness

Beyond medical readiness, HPT-resourced brigades demonstrated meaningful and sustained advantages in physical fitness, body composition compliance, and marksmanship performance—three pillars of operational readiness and warfighting capability. These domains reflect not only physiological preparedness, but also the effectiveness of unit-level training, discipline, and recovery infrastructure, all of which were enhanced by the presence of embedded H2F experts.

*ACFT:* By FY2023, HPT brigades scored 12 points higher on average and had 18% lower odds of failure (OR 0.8191). Tailored strength programs, movement coaching, and injury mitigation drove these gains. In addition to mean improvements, variability in performance also declined over time. Both HPT and control brigades demonstrated substantial reductions in ACFT confidence intervals by FY2023, suggesting increased standardization and reliability in test outcomes across the force—though the effect was more pronounced in HPT units.

*BCP:* Even amid force-wide BCP failures, HPT brigades maintained significantly lower odds of noncompliance (OR 0.9390). On-site dietitians and lifestyle coaching provided individualized support consistent with performance nutrition best practices [156, 157]. Modest CI reductions were also observed in BCP outcomes for both groups, reinforcing the stabilizing effect of data-informed policy change.

*RMQ:* HPT brigades were 1.60 times more likely to qualify expert (OR 1.5985) and 28.1% less likely to fail RMQ. This reflects integrated training environments where strength and conditioning, cognitive focus training, and mental rehearsal are synergized [158, 159].

### Significance of Findings

This evaluation offers compelling empirical evidence that embedded HPTs drive statistically significant, operationally meaningful, and fiscally defensible improvements across readiness, cost avoidance, and performance domains. These results represent not incremental, change but rather, structural transformation in how the Army preserves combat power, restores deployable strength, and maximizes return on human capital.

*National defense and operational overmatch*: MSKIs remain the Army’s leading non-battle readiness threat, responsible for more than 10 million lost duty days and more than half of all disability discharges each year [6–10]. HPT-resourced brigades demonstrated significant reductions in MSKI referrals and profile burden. Since out-of-network referrals cost $4500–11,000 per case [6, 7, 9, 133, 135], scaling this reduction across 100,000 fewer referrals could yield $660 + million in annual cost avoidance. These results directly advance Army People Strategy goals to reduce preventable attrition and enhance deployable strength [160].

*Soldier quality of life and retention*: HPTs reduce barriers to early engagement with physical and BH services—predictors of separation, retention, and suicide risk [67, 161, 162]. The presence of embedded mental readiness specialists likely contributed to reductions in both short- and long-duration BH and SA profiles, despite higher baseline rates in these units. These behavioral outcomes are not only readiness metrics but also quality-of-life indicators. Longitudinal evidence shows soldiers with embedded access and care trust are more likely to stay in service and less likely to escalate into crisis [163, 164].

*Resource efficiency and readiness resilience*: The cost of replacing a separated soldier—including recruiting, onboarding, and training—ranges from $78,000 to $130,000 + depending on specialty and pipeline length [45, 151, 152]. By preventing MSKI- and BH-related separation risks, HPTs can preserve trained personnel and reduce reaccession burden. These findings build upon Clifton et al. and Fisher et al. [65, 165], who modeled strong returns for preventative readiness interventions. Knapik et al. and Molloy et al. [10, 36, 166, 167] similarly confirmed that embedded prevention reduces training loss, downstream disability, and reconditioning burden. Improved RMQ scores in HPT brigades further reflect enhanced motor control, visual focus, and stress regulation—key elements of combat effectiveness [168, 169]. Together, these improvements indicate that embedded HPTs do not merely prevent injury, they optimize the physiological and psychological foundations of operational performance.

### Mechanisms and Operational Implications

The observed HPT effects likely do not reflect isolated program activities, but a unified set of interdisciplinary mechanisms operating across the soldier lifecycle. These mechanisms—proximal care, adaptive training, cognitive readiness, and organizational enablement—are grounded in exercise science, behavioral health, and organizational psychology. Together, they reshape readiness infrastructure from reactive to proactive and anticipatory.

*Proximal SME access*. Daily access to embedded physical therapists, athletic trainers, registered dietitians, strength and conditioning coaches, and behavioral health educators compresses the time from injury or need onset to care [43, 170, 171]. This model aligns with Gabbett’s [59] “first responder rehabilitation,” a strategy that intercepts minor injuries before they evolve into chronic dysfunction. In this study, significant reductions in both short- and long-duration MSKI profiles, including > 90-day cases, were observed in HPT brigades—despite higher baseline injury burden—suggesting systemic prevention and early intervention.

*Mental readiness and early engagement*: Mental readiness providers (e.g., cognitive performance specialists, occupational therapists, chaplains) embedded in HPTs extend BH support into the daily rhythm of training, enabling low-threshold access, early detection, and trust-based engagement. Unlike referral-based models, soldiers interact with SMEs during normal operations, which often destigmatizes care and facilitates earlier disclosure of stress or risk behaviors [172–174] These dynamics likely contributed to the significant reductions in BH and SA profiles observed in HPT brigades. Evidence from both military and civilian contexts confirms that such integration increases care utilization and adherence while reducing crisis escalation [175–177].

*Load management and autoregulated periodization*: HPTs directly manage one of the most modifiable determinants of MSKI risk: training load. Strength coaches, athletic trainers, and rehabilitation specialists guide autoregulated, periodized training adapted to each unit’s tempo, mission set, environmental conditions, and soldier readiness [178, 179]. Their interventions prevent overuse injuries and enable safe return to duty. Research shows that acute workload spikes elevate injury risk [180–182]. Embedded teams help units taper workloads during transitions, prevent overuse injury, and guide safe reentry for profiled soldiers [145, 180]. Importantly, these interventions are supported by high-level evidence. A meta-analysis by Lauersen et al. [58] demonstrated that structured strength training reduced sports injuries by more than two-thirds (RR 0.315; 95% CI 0.207–0.480), including substantial reductions in overuse injuries. These effects were consistent across populations and showed no significant heterogeneity. This reinforces strength training as one of the most effective injury prevention strategies available, and supports the observed reductions in MSKI profiles and performance gains among HPT brigades in this study [60, 64, 183].

*Integrated recovery and cognitive–physical optimization*: Military performance demands integrated cognitive and physical functioning. By coordinating recovery practices across sleep, stress, nutrition, and movement, HPTs optimize the neuromuscular and cognitive systems underlying marksmanship and combat fitness. Improvements in expert-level RMQ observed in this study align with this mechanism. Recovery protocols—sleep hygiene education, fatigue monitoring, and stress regulation—are embedded within unit routines [184, 185]. Sleep coaching and BH support also address hormonal and immune dysregulation that impairs recovery and contributes to re-injury risk [186, 187]. These integrated interventions likely contributed to reduced BCP failure rates and shortened return-to-duty timelines in HPT brigades [188, 189].

*Organizational climate and leadership enablement*: HPTs also function as force multipliers by extending the decision-making bandwidth of command teams. Through embedded presence and continuous data sharing, HPTs enable leaders to identify risk trends, adjust training, and prioritize interventions before readiness degrades [190, 191]. Organizational science affirms that units with anticipatory leadership and access to actionable performance data outperform those that rely on lagging indicators [192, 193]. In this evaluation, units with HPTs exhibited not only clinical improvements but also operational gains, supporting the role of HPTs in readiness governance. Moreover, HPT integration reinforces performance norms at the unit level, increasing perceived organizational support and fostering a high-performance culture [194, 195].

### Implications for Military Readiness and Policy

The demonstrated ROI from embedded HPTs carries direct implications for military readiness, force generation models, and defense health policy. Far from being isolated unit-level successes, the results suggest that HPTs offer a scalable and fiscally credible solution to longstanding systemic challenges in the DoD.

*Operational readiness and force generation*: HPTs improved MSKI and BH outcomes while restoring deployability—potentially generating more than $5.28 billion in annual force-wide savings. With MSKI burden estimated at $3.7 billion annually, these results validate HPTs as both clinically effective and economically dominant readiness multipliers.

*Defense health reform and financial stewardship*: HPTs align with Military Health System (MHS) reform priorities, including TRICARE restructuring and value-based care [139]. Like VA reforms, embedded teams reduce costly specialty care through proactive, localized intervention [196].

*Strategic workforce and force structure*: Avoided separations from MSKI, BH, and ACFT/BCP failure reduce recruiting and training burden—saving $78,000–130,000 per soldier [197, 198]. Whitley et al. [45] proposed an Active/Reserve hybrid force optimization model. HPT expansion—via embedded or hub-and-spoke delivery—could provide scalable readiness support across both components.

*Policy relevance and congressional oversight*. As defense oversight increasingly emphasizes outcomes-based investment, HPTs offer a quantifiable, doctrine-aligned readiness solution. They deliver tangible returns on prevention, retention, and combat performance, reinforcing modernization priorities under fiscal constraint. Broader institutionalization of HPTs would represent a strategic upgrade in how the Army sustains force health and readiness.

### Scientific and Programmatic Contribution

This evaluation makes a significant contribution to military science, defense health economics, and implementation research. It is the first large-scale, quasi-experimental analysis to quantify both operational and fiscal returns from embedded HPTs across the U.S. Army. Using a presence-based DiD framework, domain-specific cost mapping, and Monte Carlo simulation, this study advances how readiness optimization is evaluated in real-world military settings.

*Methodological advancements:* This study introduces four methodological innovations: (1) policy-driven, presence-based, intent-to-treat DiD modeling that enhances generalizability; (2) validated cost-per-case inputs from DoD, RAND, DHA, and peer-reviewed literature that improve fiscal credibility; (3) Monte Carlo simulation enables probabilistic inference; and (4) stratified extrapolation accounts for Active and Reserve Component structural differences, improving force-wide projections.

*Scientific crosswalk:* In human performance, this evaluation supports the effectiveness of embedded multidisciplinary teams. HPTs improved MSKI, BH, SA, ACFT, and RMQ outcomes, providing real-world evidence for integrated performance systems [199, 200]. In organizational science, the findings affirm constructs such as perceived organizational support [194], social learning and modeling [201–203], and the impact of unit climate on help-seeking behavior [204]. In health services research, this study demonstrates that short-horizon ROI modeling using validated, domain-specific costs can drive value-based planning in decentralized, operational contexts [109, 205]. The design aligns with best practices in pragmatic evaluation and fiscal accountability [107, 206]. In implementation science, this study offers a replicable model for formative program evaluation. By linking HPT presence to downstream readiness differentials—across multiple outcome domains with convergent directionality and statistical significance—it demonstrates the aggregate effect of embedded systems. This framework supports application in other complex, decentralized systems such as education, law enforcement, or public health [177, 207].

*Programmatic value and DoD-wide transferability*: Beyond academic relevance, this evaluation informs Army planning, programming, budgeting, and execution processes by demonstrating the measurable fiscal and operational impact of HPTs. It provides a reproducible ROI estimation architecture that supports bottom-up resource justification. These methods are transferable across services and agencies, offering a blueprint for outcome-driven modernization under the DoD’s evolving readiness, value-based care, and people-first strategies.

### Limitations

This evaluation was designed to be methodologically conservative and operationally grounded. While the findings are robust, several limitations must be acknowledged to guide interpretation, inform future research, and refine implementation strategies.

*Nonrandomized design and baseline imbalance*: HPT brigades were not randomly assigned and started with worse MSKI, BH, and SA profile outcomes. While DiD modeling adjusts for baseline imbalance and secular trends [208, 209], unmeasured confounders (e.g., leadership dynamics) may still influence outcomes. However, consistent, directional improvement across multiple domains supports a true treatment effect [79–81, 210].

*Conservative scope and undervaluation*: The analysis excluded long-horizon, intangible, and system-spillover benefits. These include VA disability avoidance, retention gains, and quality-adjusted life years. Prior research suggests these effects could raise per-soldier lifetime value by $100,000 + [68, 72, 146, 211].

*Extrapolation to the reserve component*: Reserve component modeling used a conservative 0.65 discount on the basis of access gaps and fielding heterogeneity. While grounded in prior evaluations, future models should incorporate fidelity-adjusted engagement metrics and component-specific delivery effects.

*Partial contamination of control group*: While this evaluation modeled HPT resourcing as a binary presence/absence variable, it is possible that a small number of control brigades implemented partial or local H2F initiatives (e.g., part-time access to SMEs, standalone pilot programs, or related wellness activities). These exposures were not formally tracked or resourced through the embedded HPT model and thus were not captured in the analytic dataset. However, any such contamination would likely dampen the observed treatment effect, biasing estimates toward the null. Therefore, the observed magnitude of outcome improvement in HPT brigades may be conservative, suggesting that the true causal effect of embedded teams could be even greater under fully controlled conditions.

*Fidelity and utilization unknowns*: Presence-based models only identify whether a brigade was resourced with an HPT—not how effectively or consistently the team was used. Variation in SME integration, soldier access, and command support likely drives heterogeneity in outcomes. Implementation science literature identifies fidelity as a primary determinant of intervention effectiveness [177, 212]. Future research should incorporate a Utilization Score (U-Score; currently under development), or similar fidelity-sensitive metric capturing exposure frequency, intensity, duration, and task-relevance to enable dose–response modeling [213, 214].

*Tier structure and ceiling effects*: Although the HPT staffing model was tiered on the basis of brigade type and operational need, the evaluation treated HPT presence as a binary variable (resourced versus control) due to limited fidelity in year-to-year staffing rates and variable implementation timelines. While tier 1 units theoretically received the most robust HPT complement, hiring lags, SME turnover, and incomplete fielding prior to FY2022 limited the reliability of tier-based modeling. Importantly, even high-performing brigades—presumed to have higher baseline readiness—showed measurable gains in ACFT pass rates, MSKI burden reduction, and RMQ performance, suggesting that HPT resourcing provided additive value across the performance spectrum.

*Static cost inputs and inflation risk*: All costs were indexed to FY2023 USD, but recruiting and separation costs are rising—up > 40% since 2013. This may understate future savings. Dynamic cost indexing and inflation-adjusted updates are recommended [129].

*Latent spillover and climate effects*: HPTs likely influence help-seeking, adherence, and readiness culture. These “amplifier effects” cannot yet be quantified [215, 216], but future mixed-methods research (e.g., social network analysis) could explore these second-order gains [216–218].

*Lack of demographic stratification:* Individual-level covariates such as sex, age, and rank were not included in the analytic model due to the deidentified, unit-level structure of the dataset. Sex-disaggregated data were only available for ACFT and BCP. As a result, potential demographic moderators of program effectiveness—particularly sex-specific injury or BH trends—could not be evaluated.

*Unmeasured turnover effects:* Annual personnel turnover was not tracked across brigades. Year-over-year changes in soldier composition, leadership, or MOS mix may introduce uncontrolled variance in outcome trajectories. While DiD modeling adjusts for secular trends and baseline imbalance, unobserved churn in personnel characteristics could dampen or obscure true effects. The fact that consistent, directional improvements were observed across outcome domains—despite potential dilution from high turnover—strengthens the inference of a real and durable treatment effect attributable to HPT resourcing.

### Future Directions

To maximize program impact, H2F modernization should evolve from expansion to precision, leveraging data infrastructure, fidelity metrics, and adaptive analytics.

*Scale embedded HPTs:* With $24.44 million in annual TEV per brigade and 100% of Monte Carlo draws above an ROI of 3.0 (in cost avoidance alone), embedded HPTs should be prioritized across the force—not as adjunct services, but as essential components of operational readiness. Staffing models should be tailored to unit structure, with ongoing fidelity/utilization tracking to optimize ROI. DoD-level policy and presidential/congressional budgets should ensure these critical assets are secured, fully implemented, and modernized across all branches of service.

*Deploy the H2F management system* (*H2FMS*): The H2FMS is the digital backbone of H2F modernization [219]. It integrates soldier-level data on training, education, assessment, and intervention across all five H2F readiness domains, enabling personalized programming, SME coordination and coaching, real-time role-based dashboards, and toolkits for measuring, understanding, and improving holistic soldier readiness. Operationally, the H2FMS expands HPT reach, supports asynchronous interventions, and enables fidelity monitoring at scale—evolving H2F into a dynamic readiness ecosystem for Total Army impact.

*Integrate wearables for real-time readiness*: Validated wearables can capture sleep quality, heart rate variability, thermal load, and musculoskeletal fatigue, generating predictive insights that allow for preemptive intervention [220]. When synchronized with the H2FMS, these metrics could enable near-real-time optimization of training intensity, recovery, and mission planning [221, 222]. While there is currently minimal evidence of significant cost–benefit for the Army procuring and deploying wearables writ large, a “bring-your-own-device” (BYOD) or targeted subgroup approach offers a low-burden solution for remote monitoring and tailored support. With appropriate cybersecurity and privacy protocols, biometric sensors could shift from luxury to become a strategic enabler of future-ready force health protection.

*Operationalize the utilization score* (*U-Score*): To move beyond presence-based metrics, the U-Score quantifies actual H2F engagement at the soldier–month level. It incorporates frequency, intensity, duration, and physiological relevance of HPT encounters, generating a continuous fidelity metric usable for predictive modeling. This enables dose–response evaluation, minimum effective thresholds, and resource targeting. Implementing the U-Score will transform H2F from a binary variable into a precision instrument for intervention optimization and cost-effectiveness modeling—aligned with modern implementation science [213, 223].

*Modernize reserve component support models*: Reserve component implementation must be deliberately designed for episodic access and geographic dispersion. Regional hubs, mobile teams, and digital platforms offer scalable solutions. Wearable integration and adaptive engagement metrics will ensure fidelity and impact even in nontraditional formats. Select high-readiness units may require hybrid or embedded support. All models should be continuously evaluated for reach, quality, and outcomes to ensure Reserve component soldiers receive full-spectrum performance support, critical to total Army readiness.

*Embed adaptive analytics and policy triggers*: H2F must evolve into a learning system. Embedded analytics, outcome deltas, and real-time ROI simulations can identify inflection points and drive data-informed resourcing. Establishing policy triggers, e.g., MSKI referral reductions or BH engagement dips, can prompt rapid, localized intervention. A central analytic cell under H2F should steward predictive model development, economic recalibration, and adaptive experimentation. This will ensure H2F remains responsive, effective, and accountable across operational environments and budget cycles.

## Concluions

This study represents the most comprehensive readiness and cost-effectiveness evaluation of embedded HPTs to date. Using a matched DiD framework across 56 brigades and validated domain-specific costs, it estimates $24.44 million in annual total economic value per brigade (ROI = 8.15:1), with > 95% of simulations exceeding 5:1. HPTs deliver reliable returns across MSKI, BH, SA, ACFT, BCP, and RMQ outcomes. They are a high-return investment that improves readiness, reduces preventable attrition, and conserves defense resources. Their impact is not speculative—it is reproducible, operationally verified, and policy relevant. Embedded HPTs do not merely reduce injuries. They shift the structure, tempo, and resilience of the Army’s readiness architecture. As the Army faces escalating operational complexity and resource constraint, embedded HPTs offer a validated, data-driven, and mission-aligned solution. They are not ancillary wellness programs. They are readiness infrastructure, strengthening the Army’s most critical weapon system—the soldier—while maximizing return on investment and preserving combat power. Their expansion is not a discretionary wellness upgrade. It is a strategic imperative for force modernization, fiscal stewardship, and warfighting capability.

## Supplementary Information

Below is the link to the electronic supplementary material.

Supplementary file1 (DOCX 33 KB)

## Declarations

### Availability of data and material

The datasets generated during and/or analyzed during the current study are not publicly available due to potential national security implications but may be available from the public affairs office pending a formal request, documented need to know requirement, verification of data security practices and authorizations, completion of a data sharing agreement, and formal review. All requests to initiate this process should be directed to U.S. Army Training and Doctrine Command Center for Initial Military Training Public Affairs Office at usarmy.jble.tradoc.mbx.cimt-pao@army.mil.
