# Supplementary material for: Evaluating the Return on Investment of U.S. Army Holistic Health and Fitness Performance Teams: A Matched Difference-in-Differences Study of Readiness and Economic Outcomes
Source: Sports Med. 2026 Feb 12;56(7):1801–31. doi: 10.1007/s40279-026-02399-3 (PMC13388362; doi:10.1007/s40279-026-02399-3)
Supplement: Supplementary file 1 — Supplementary file1 (DOCX 33 KB) [file 40279_2026_2399_MOESM1_ESM.docx]

Online Resources 1

Article Title:

*Evaluating the Return on Investment of U.S. Army Holistic Health and Fitness Performance Teams: A Matched Difference-in-Differences Study of Readiness and Economic Outcomes*

Journal Name:

*Sports Medicine*

Author Names:

Andrew G Thompson, Manoj Subedi, Alex E Morrow, Chance L Smith, and Kevin A Bigelman

Affiliation:

Center for Initial Military Training, United States Army Training and Doctrine Command

Corresponding Author:

Andrew G Thompson, PhD, MS

Andrew.G.Thompson15.civ@army.mil

ORCID: 0000-0002-2251-1384

Supplemental Table 1. H2F Performance Team Return on Investment Metrics

| **HPT ROI Metric** | **Data Element(s)** | **Response Time** | **Objective** |
| --- | --- | --- | --- |
| ACFT | Pass/Fail Rates/Counts | Early | Increase Pass |
| Attrition from Injury | Rates/Counts | Early | Decrease |
| IET Attrition | Rates/Counts | Early | Decrease |
| Body Composition | Pass/Fail Rates/Counts | Early | Decrease Fail |
| Limited Duty Profiles | MSKI Rates/Counts  MSKI > 90 Days Rates/Counts  BH Rates/Counts  BH > 90 Days Rates/Counts | Early  Early  Early  Early | Decrease  Decrease  Decrease  Decrease |
| Medical Cost Avoidance | Monetary Savings | Early | Increase |
| Out of Network Care Referrals | MSKI Referral Rates/Counts | Early | Decrease |
| Quality of Life | Soldier Readiness Surveys | Early | Increase |
| Substance Abuse Profiles | Rates/Counts | Early | Decrease |
| Weapons Qualification | Pass/Fail Rates/Counts  Expert Rates/Counts | Early  Early | Increase Pass  Increase |
| Functional Training | School Graduation Rates/Counts | Moderate | Increase |
| First Term Reenlistment | Rates/Counts | Moderate | Increase |
| First Term Attrition | Rates/Counts | Moderate | Decrease |
| Suicide Attempts | Rates/Counts | Moderate | Decrease |
| Suicides | Rates/Counts | Moderate | Decrease |
| Non-Deployability | Rates/Counts | Moderate | Decrease |
| Permanent Profiles | Rates/Counts | Long | Decrease |

**Note** HPT = H2F Performance Team; ACFT = Army Combat Fitness Test; IET = Initial Entry Training; BCP = Body Composition Program; MSKI = musculoskeletal injury; BH = behavioral health; SA = substance abuse; ROI = return on investment

Response time windows (early, moderate, long) are categorized according to anticipated biological, behavioral, and organizational adaptation timelines, consistent with military epidemiology, training science, and implementation research.

- *Early response metrics (1–3 years post-resourcing):* Outcomes expected to change rapidly with HPT implementation, primarily because they reflect short-cycle biological adaptation, direct behavioral modification, or administrative tracking. These include improvements in physical test performance (ACFT), reductions in near-term attrition (IET, injury-related), decreased BCP failure, reduced MSKI and BH limited duty profiles, lower SA profiles, reduced specialty referrals, and immediate monetary savings from decreased treatment burden. Such outcomes are sensitive to program initiation and can be observed within a few training cycles or fiscal years.
- *Moderate response metrics (3–5 years):* Outcomes that require sustained exposure to interdisciplinary practice, culture change, and system-level reinforcement. Examples include reenlistment rates, attrition beyond IET, suicide attempts and completions, reductions in non-deployability, and graduation rates from physically demanding training schools. These outcomes are influenced by cumulative improvements in physical readiness, resilience, and behavioral health support, which manifest after multiple cohorts have trained and deployed under HPT support.
- *Long-term response metrics (≥ 5 years):* Outcomes reflecting enduring musculoskeletal and behavioral health protection, chronic condition prevention, and retention benefits. Permanent profiles and disability deferrals accumulate slowly and require several years of consistent intervention to demonstrate measurable reductions at population scale. These lagging indicators are critical to capturing the full value of H2F Performance Teams, but are not expected to move substantially in the earliest phases of implementation.

This temporal classification provides a framework for interpreting observed effects: early metrics signal initial program success, moderate metrics indicate consolidation of practice and cultural change, and long metrics capture durable, force-wide return on investment.

Supplemental Table 2. Musculoskeletal Injury Outcomes Before and After H2F Performance Team Resourcing

| **ROI Metric** | **FY** | **N** | **χ ^2^** | ***p*** | **FE** | **Result** |
| --- | --- | --- | --- | --- | --- | --- |
| Musculoskeletal  Injury  Profiles | 2019 | 201727 | 38.443 | < .0001 | < .0001 | HPT Higher |
|  | 2020 | 199303 | 20.670 | < .0001 | < .0001 | HPT Higher |
|  | 2021 | 210288 | 12.883 | .0003 | .0002 | HPT Lower |
|  | 2022 | 208537 | 13.688 | .0002 | < .0001 | HPT Higher |
|  | 2023 | 201187 | 22.472 | < .0001 | < .0001 | HPT Higher |
| Musculoskeletal  Injury  Profiles > 90 Days | 2019 | 36846 | 11.091 | .0009 | .0005 | HPT Higher |
|  | 2020 | 35042 | 0.557 | .4553 | NS | NS |
|  | 2021 | 45316 | 6.254 | .0124 | < .0001 | HPT Higher |
|  | 2022 | 44289 | 15.120 | < .0001 | < .0001 | HPT Higher |
|  | 2023 | 51846 | 0.602 | .4378 | NS | NS |
| Musculoskeletal  Injury  Referrals Out for Civilian Care | 2019 | 36846 | 48.188 | < .0001 | < .0001 | HPT Higher |
|  | 2020 | 35042 | 140.846 | < .0001 | < .0001 | HPT Higher |
|  | 2021 | 45316 | 87.657 | < .0001 | < .0001 | HPT Higher |
|  | 2022 | 44289 | 22.582 | < .0001 | < .0001 | HPT Lower |
|  | 2023 | 51846 | 1785.681 | < .0001 | < .0001 | HPT Lower |

**Note** This table presents the musculoskeletal-related readiness for duty Chi-square frequency distribution and Fisher’s Exact (FE) results across fiscal years (FY). In FY2019, neither cohort was resourced with an H2F Performance Team (HPT). Resourcing began in FY2020 and reached full operational capacity (at least 75% staffing) by the end of FY2021. For each FY, the resulting relationship between each outcome is described in terms of higher or lower probability of occurrence for the cohort with (or receiving) HPTs. NS = not significant

Supplemental Table 3. Behavioral Health Outcomes Before and After H2F Performance Team Resourcing

| **ROI Metric** | **FY** | **N** | **χ ^2^** | ***p*** | **FE** | **Result** |
| --- | --- | --- | --- | --- | --- | --- |
| Behavioral Health  Profiles | 2019 | 201727 | 38.553 | < .0001 | < .0001 | HPT Higher |
|  | 2020 | 199303 | 22.692 | < .0001 | < .0001 | HPT Higher |
|  | 2021 | 210288 | 11.373 | .0007 | .0004 | HPT Higher |
|  | 2022 | 208537 | 2.242 | .1343 | NS | NS |
|  | 2023 | 201187 | 9.883 | .0017 | .0009 | HPT Lower |
| Behavioral Health  Profiles > 90 Days | 2019 | 4868 | 46.923 | < .0001 | < .0001 | HPT Higher |
|  | 2020 | 4740 | 12.214 | .0005 | .0003 | HPT Higher |
|  | 2021 | 6167 | 36.608 | < .0001 | < .0001 | HPT Higher |
|  | 2022 | 6400 | 3.900 | .0483 | .0256 | HPT Higher |
|  | 2023 | 6995 | 12.241 | .0005 | .0003 | HPT Lower |
| Substance  Abuse  Profiles | 2019 | 201727 | 105.233 | < .0001 | < .0001 | HPT Higher |
|  | 2020 | 199303 | 52.870 | < .0001 | < .0001 | HPT Higher |
|  | 2021 | 210288 | 0.123 | .7253 | NS | NS |
|  | 2022 | 208537 | 15.123 | < .0001 | < .0001 | HPT Lower |
|  | 2023 | 201187 | 631.116 | < .0001 | < .0001 | HPT Lower |

**Note** This table presents the behavioral health-related readiness for duty Chi-square frequency distribution and Fisher’s Exact Test (FE) results across fiscal years (FY). In FY2019, neither cohort was resourced with an H2F Performance Team (HPT). Resourcing began in FY2020 and reached full operational capacity (at least 75% staffing) by the end of FY2021. For each FY, the resulting relationship between each outcome is described in terms of higher or lower probability of occurrence for the cohort with (or receiving) HPTs. NS = not significant

Supplemental Table 4. Performance Readiness Before and After H2F Performance Team Resourcing

| **ROI Metric** | **FY** | **N** | **χ ^2^** | ***p*** | **FE** | **Result** |
| --- | --- | --- | --- | --- | --- | --- |
| Failing  Body Composition Program | 2019 | 114493 | 68.324 | < .0001 | < .0001 | HPT Lower |
|  | 2020 | 51767 | 17.725 | < .0001 | < .0001 | HPT Lower |
|  | 2021 | 129610 | 44.715 | < .0001 | < .0001 | HPT Lower |
|  | 2022 | 135110 | 62.207 | < .0001 | < .0001 | HPT Lower |
|  | 2023 | 227629 | 15.503 | < .0001 | < .0001 | HPT Lower |
| Passing  Army Combat  Fitness Test | 2019 | 212 | .020 | .8889 | NS | NS |
|  | 2020 | 22456 | 10.773 | .0010 | .0002 | HPT Higher |
|  | 2021 | 117140 | 57.332 | < .0001 | < .0001 | HPT Higher |
|  | 2022 | 118360 | 74.478 | < .0001 | < .0001 | HPT Higher |
|  | 2023 | 128174 | 33.358 | < .0001 | < .0001 | HPT Higher |
| Passing Rifle Marksmanship Qualification | 2019 | 126637 | .792 | .3735 | NS | NS |
|  | 2020 | 16801 | 86.268 | < .0001 | < .0001 | HPT Higher |
|  | 2021 | 32698 | 0.021 | .0885 | NS | NS |
|  | 2022 | 98539 | 13.335 | .0003 | .0001 | HPT Higher |
|  | 2023 | 127465 | 10.174 | .0014 | .0010 | HPT Higher |
| Passing Rifle Marksmanship Qualification as an Expert | 2019 | 126637 | 275.904 | < .0001 | < .0001 | HPT Higher |
|  | 2020 | 16801 | 65.857 | < .0001 | < .0001 | HPT Higher |
|  | 2021 | 32698 | 105.200 | < .0001 | < .0001 | HPT Higher |
|  | 2022 | 98539 | 326.151 | < .0001 | < .0001 | HPT Higher |
|  | 2023 | 127465 | 1321.291 | < .0001 | < .0001 | HPT Higher |

**Note** This table presents the Performance Readiness Chi-square frequency distribution and Fisher’s Exact Test (FE) results across fiscal years (FY). In FY2019, neither cohort was resourced with an H2F Performance Team (HPT). Resourcing began in FY2020 and reached full operational capacity (at least 70% staffing) by the end of FY2021. For each FY, the resulting relationship between each outcome is described in terms of higher or lower probability of occurrence for the cohort with (or receiving) HPTs. NS = not significant
